# A pyroptosis‐related lncRNA signature in bladder cancer

**DOI:** 10.1002/cam4.5344

**Published:** 2022-10-13

**Authors:** Peng Wang, Zhiqiang Wang, Liping Zhu, Yilan Sun, Leandro Castellano, Justin Stebbing, Zhentao Yu, Ling Peng

**Affiliations:** ^1^ Department of Medical Oncology Yidu Central Hospital of Weifang Weifang China; ^2^ Department of Urology Shouguang Hospital of Traditional Chinese Medicine Shouguang China; ^3^ Department of Medical Oncology Shouguang Hospital of Traditional Chinese Medicine Shouguang China; ^4^ Department of Respiratory Disease Zhejiang Provincial People's Hospital, Affiliated People's Hospital, Hangzhou Medical College Zhejiang China; ^5^ Department of Biochemistry, School of Life Sciences University of Sussex Brighton UK; ^6^ Division of Cancer, Department of Surgery and Cancer Imperial College London London UK; ^7^ Department of Biomedical Sciences Anglia Ruskin University Cambridge UK; ^8^ Department of Thoracic Surgery, National Cancer Center, National Clinical Research Center for Cancer Cancer Hospital and Shenzhen Hospital, Chinese Academy of Medical Sciences and Peking Union Medical College Shenzhen China

**Keywords:** bladder cancer, lncRNA, prognostic signature, Pyroptosis, tumor immune microenvironment

## Abstract

**Purpose:**

Pyroptosis, a type of programmed cell death, is implicated in the tumorigenesis, development and migration of cancer, which can be regulated by long non‐coding RNAs (lncRNAs). Our research aimed to investigate the prognostic role of pyroptosis‐related lncRNAs and the relationship to the tumor immune microenvironment through bioinformatics analysis.

**Methods:**

The clinical and RNA‐sequencing data of bladder cancer patients were downloaded from The Cancer Genome Atlas (TCGA). And 412 bladder cancer subjects with clinical information were divided into training and testing cohort. And 52 reported pyroptosis‐related genes were used to screen pyroptosis‐related lncRNAs. A pyroptosis‐related lncRNA signature was constructed based on Cox regression analyses.

**Results:**

A 9‐pyroptosis‐related‐lncRNA signature was identified to separate patients with bladder cancer into two groups. The prognosis of bladder cancer patients in the high‐risk group was significantly inferior compared with those in the low‐risk group. Risk scores were validated to develop an independent prognostic indicator based on multivariate Cox regression analysis. Receiver operating characteristic curve (ROC) analysis examined the signature on overall survival. The area under time‐dependent ROC curve (AUC) at 1‐, 3, and 5‐years measured 0.747, 0.783, and 0.768, respectively. Analysis of the immune landscape and PD‐L1 expression showed that PD‐L1 is upregulated in the high‐risk group. The immunocyte subtypes of the two groups were different.

**Conclusion:**

A novel pyroptosis‐related lncRNA signature was identified with prognostic value for bladder cancer patients. Pyroptosis‐related lncRNAs have a potential role in cancer immunology and may serve as prognostic or therapeutic targets.

## Graphical abstract

The clinical and RNA‐sequencing data of bladder cancer patients were downloaded from The Cancer Genome Atlas (TCGA). Pyroptosis‐related genes between tumor tissues and normal were analyzed. The signature from 25 pyroptosis‐related IncRNAs was screened. A pyroptosis‐related lncRNA signature was constructed based on Cox regression analyses. The prognosis of bladder cancer patients in the high‐risk group was significantly inferior compared with those in the low‐risk group. Receiver operating characteristic curve (ROC) analysis examined the signature on overall survival. Analysis of the immune landscape and PD‐L1 expression showed that PD‐L1 is upregulated in the high‐risk group. The immunocyte subtypes of the two groups were different. Pyroptosis‐related lncRNAs have a potential role in cancer immunology and may serve as prognostic or therapeutic targets.

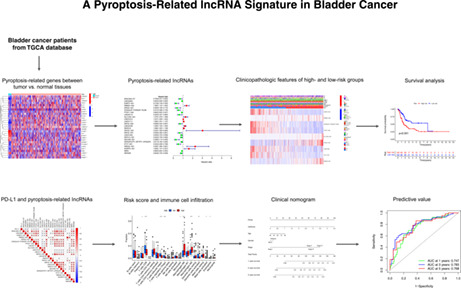

## 1 INTRODUCTION

Bladder cancer is the ninth common malignancy in the world and the most commonly diagnosed cancer of the urinary system.^1^, ^2^ About one quarter of bladder cancer patients have muscle‐invasive or metastatic disease^3^, ^4^ and approximately half of the patients with muscle‐invasive disease will relapse or metastasize after surgery.^5^, ^6^ Chemotherapy and immune checkpoint inhibitors (ICIs) have provided survival benefits for patients with metastatic bladder cancer, but clinical outcomes have varied among patients receiving standard therapy.^7^ In order to improve bladder cancer survival, many are investigating biomarkers to inform prognosis and treatment response.

Chemotherapeutic drugs inhibit cell proliferation and induce programmed cell death (PCD), thus exerting anti‐tumor effects. However, cancer cells can become resistant to PCD during chemotherapy, promoting recurrence. Previously, apoptosis was regarded as the main type of PCD, however, cancer cells can escape cell death through various mechanisms.^8^ Pyroptosis, an emerging type of PCD, can be induced by cancer chemotherapy.^9^, ^10^, ^11^ Targeting other forms of PCD provides a potential strategy to overcome chemotherapy resistance. Pyroptosis release danger‐associated signaling molecules and cytokines, which in turn activate the immune system.^12^ Pyroptosis has a proinflammatory effect, which is related to the regulation of the tumor immune microenvironment. Expression of gasdermin D (GSDMD), executor of pyroptosis, is required for effector CD8+ T cell responses.^13^ The role of pyroptosis in the anti‐tumor function of natural killer (NK) cells has also been shown in a recent study.^14^

Long non‐coding RNAs (long ncRNAs, lncRNAs) are a type RNA, with lengths exceeding 200 bps that do not code for proteins.^15^ LncRNAs are comprised of heterogeneous group of transcripts that regulate gene expression. They are involved in various diseases via multiple mechanisms, such as transcriptional, post‐transcriptional, and epigenetic modifications. Recent evidence has revealed that lncRNAs are regulators of cell pyroptosis.^16^, ^17^, ^18^, ^19^

The regulation of lncRNAs in pyroptosis is also involved in various cancer types. For example, knockdown of SNHG7 (small nucleolar RNA host gene 7), a lncRNA gene, increased the expression levels of NLRP3 (NOD‐, LRR‐ and pyrin domain‐containing protein 3) and interleukin‐1β, resulting in pyroptosis.^20^ Knockdown of MEG3 could reverse the inhibition of cisplatin on tumor growth and metastasis thorough NLRP3/caspase‐1/GSDMD pathway‐mediated pyroptosis.^21^ Based on the importance of lncRNAs, further research is warranted.^22^

The current study aimed to explore pyroptosis‐related lncRNAs in bladder cancer, which could provide evidence on the signaling pathways implicated in pyroptosis in bladder cancer and link to patient prognosis. In addition, we also analyze the relationship of pyroptosis and the tumor immune microenvironment which could provide information for use of immunotherapy in bladder cancer.

## 2 MATERIALS AND METHODS

### 2.1 Data source

**FIGURE 1:**
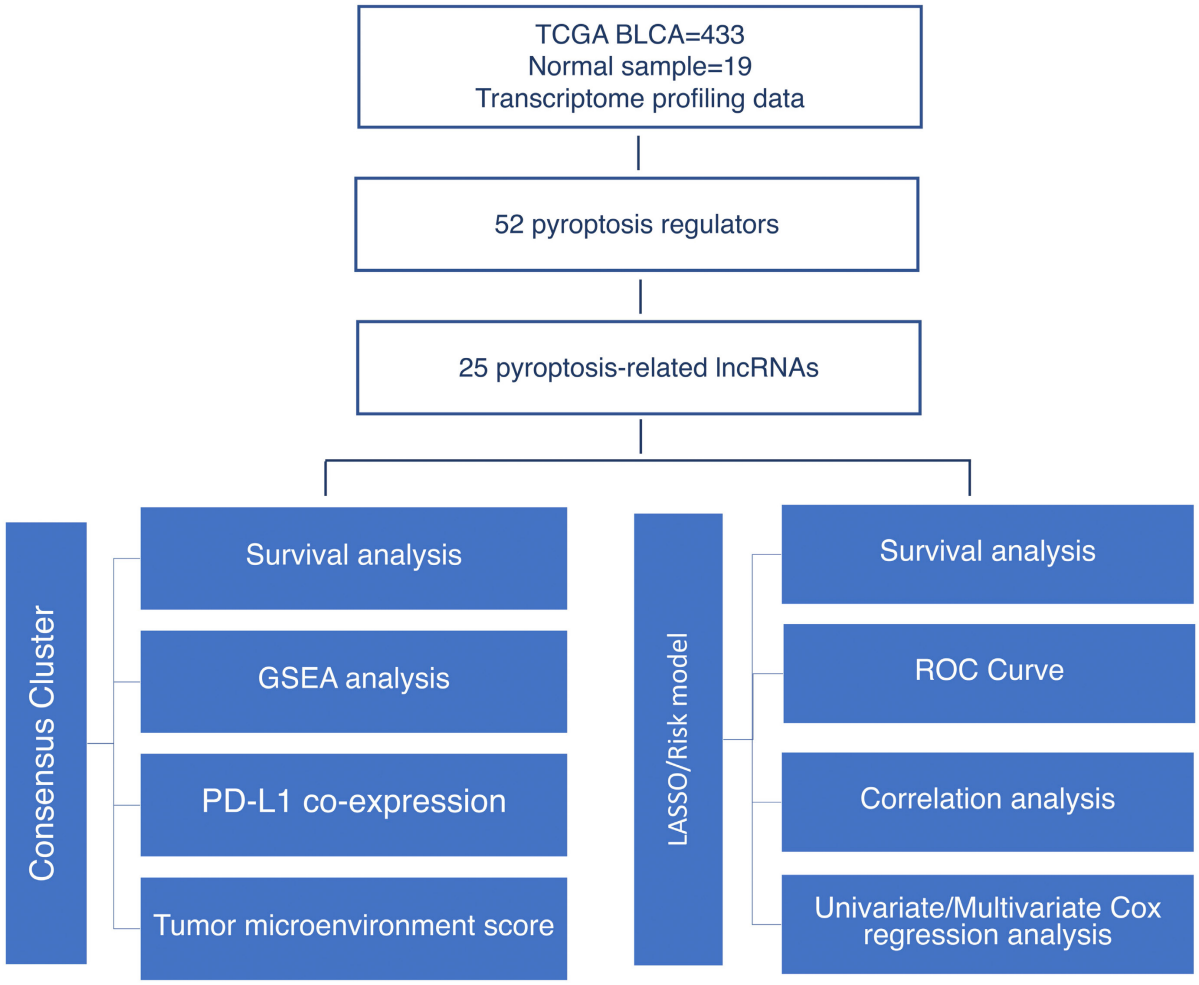
Flow diagram of our study.

Data were retrieved from TCGA database (http://cancergenome.nih.gov/) on August 02, 2021: RNA‐seq transcriptome and clinicopathological data from 433 and 412 bladder cancer (BLCA) patients, respectively; RNA‐seq transcriptome from 19 healthy controls. The different numbers of RNA‐seq transcriptome of clinical patients were matched. FPKM (fragment per kilobase of exon model per million) data were downloaded for differential analysis. The 412 BLCA patients were separated into a training and a testing cohort in a 1:1 ratio using the “caret” R package. Then, tumor mutation burden (TMB) per megabase was calculated. The flow chart of our study was illustrated in Figure 1.

### 2.2 Differentially expressed pyroptosis‐related genes

The information of 52 pyroptosis‐related genes (PRGs) were obtained from previous literature^23^, ^24^, ^25^, ^26^, ^27^, ^28^, ^29^, ^30^, ^31^, ^32^ (Table S1). To observe differences in pyroptosis‐related genes and their co‐expressed lncRNAs between bladder cancer and control groups, heatmaps and boxplots were generated by using the “limma” package, and univariate Cox regression was performed to screen the signature in pyroptosis‐related lncRNAs. The Search Tool for the Reval of Interacting Genes (STRING) was used to construct a protein–protein interaction (PPI) network for 52 PRGs.

### 2.3 Detection of regulators of pyroptosis and co‐expression lncRNAs

Expression of the 52 pyroptosis regulators was analyzed. Coexpression analysis was then performed, and the filter conditions are “*p*valueFilter = 0.001” and “correlation coefficient = 0.4”. The “igraph” R package was used to obtain the expression data co‐expression network for lncRNAs. The differences in pyroptosis‐related regulators and their co‐expressed lncRNAs between bladder cancer and control groups were shown. The signature of 25 selected pyroptosis‐related lncRNAs was screened using univariate Cox regression.

### 2.4 Consensus clustering

The patients were assigned into two categories using “ConsensusClusterPlus” package with 1000 iterations and the resample rate of 80%. The algorithm was 1000 permutations for random sampling. Overall survival of the two clusters was compared. The functions and downstream access of the two clusters were explored using gene set enrichment analysis (GSEA).

### 2.5 Construction of the prognostic signature

Least absolute shrinkage and selection operator (LASSO) regression analysis was used to establish a pyroptosis‐related lncRNAs‐associated prognostic model (PLPM). The risk score was calculated as below:

$$\text{The risk score}=\sum_{i=1}^{n}\;{\text{Coef}}_{i}\times {\text{Expr}}_{i}$$

where Expr_*i*_ represents the expression level of gene *i*, and coef_*i*_ indicates the regression coefficient of gene *i* in the signature. The risk score of each sample was calculated. Patients were separated into high‐ and low‐risk groups based on the median risk score.

### 2.6 Evaluation of prognostic value of the signature

The difference of overall survival between high‐ and low‐risk groups in the training and testing cohorts was investigated. ROC curves were implemented, and the AUC was calculated. Cox regression models were used to prove whether the risk score is an independent factor for prognosis.

### 2.7 Genomic alteration and co‐expression level of PD‐L1

PD‐L1 mutations and deletions copy number alterations (CNAs) in bladder cancer patients were analyzed from the cBioPortal tool (http://cbioportal.org). The OncoPrint displayed genetic alterations of PD‐L1 in bladder cancer samples. The association between PD‐L1 expression and pyroptosis‐related lncRNAs was depicted using “corrplot” package.

### 2.8 Evaluation of immune infiltration

The immune‐scores in the bladder cancer patients were calculated via the ESTIMATE algorithm. The fraction scores in each tumor sample for 22 immune cell subtypes were identified by CIBERSORT (cell type identification by estimating relative subtypes of RNA transcripts). The algorithm was 1000 permutations, and samples with *P* < 0.05 were incorporated. The immune infiltration levels were compared.

### 2.9 Statistical analysis

The statistical analyses were performed by R software (4.0.3). The prognostic model was constructed in LASSO Cox regression analysis. A difference of p < 0.05 indicated statistical significance. In some cases, “*p* < 0.05” gave too many results, and in these settings, “*p* < 0.01” was used as the filter factor.

## 3 RESULTS

### 3.1 DEGs between tumor and normal and tissues

**FIGURE 2:**
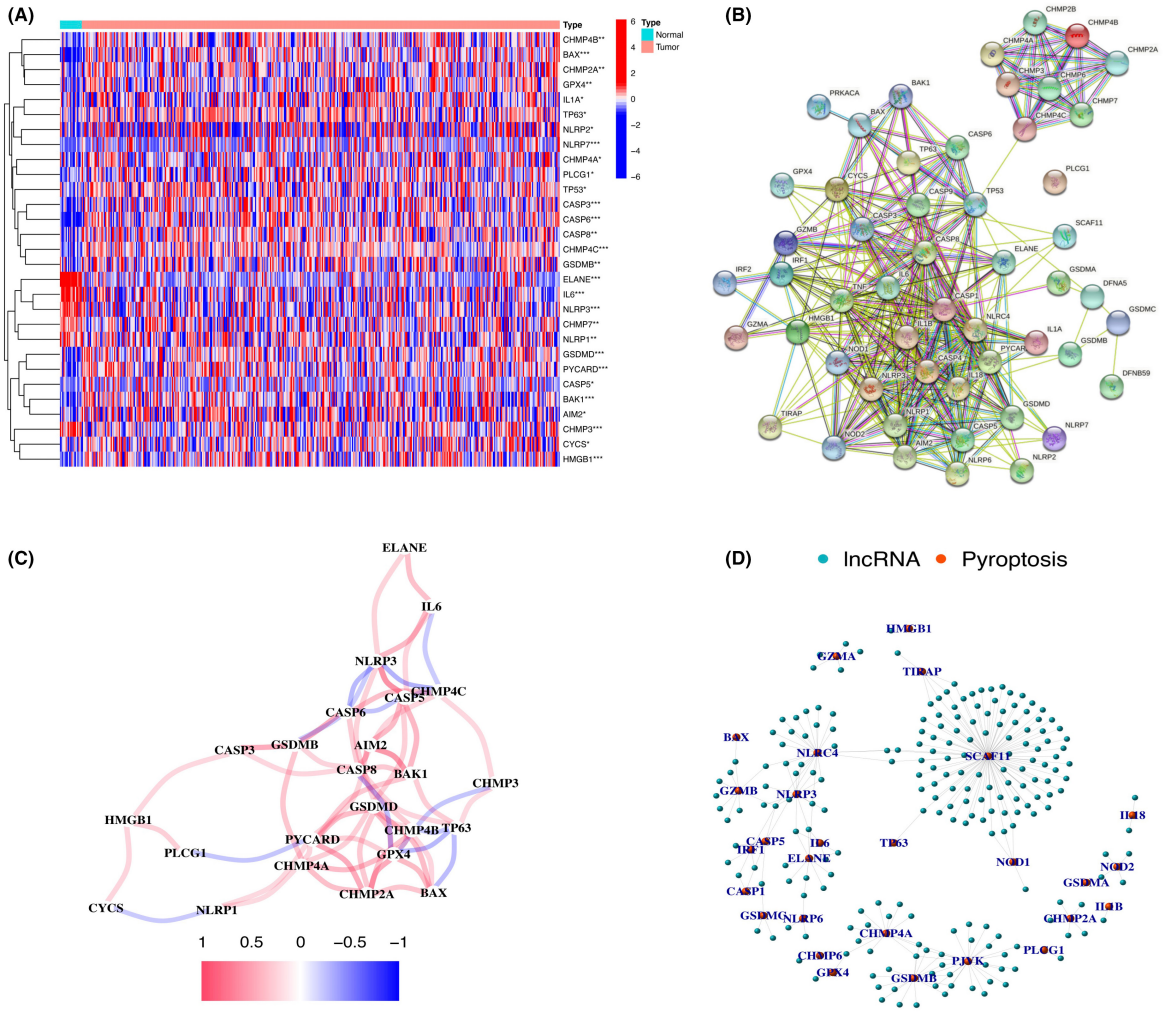
Expression and interaction of PRGs. (A) Heatmap of the PRGs between tumor tissues (T, red) and normal (N, blue). Green indicates low expression level, and red represents high expression level. *p < 0.05; **p < 0.01; ***p < 0.001. (B) The interactions of the PRGs were shown in PPI network (interaction score = 0.40). (C) Correlation network of PRGs. Red line indicates positive correlation, and blue line represents negative correlation.

The expressions of 52 pyroptosis‐related genes were compared in TCGA database from 414 tumor and 19 normal tissues. And 29 DEGs were identified. Among them, six genes (ELANE, IL6, NLRP1, NLRP3, CHMP7, and CHMP3) were downregulated while 23 other genes (GPX4, CHMP2A, CHMP4A, CHMP4B, CHMP4C, BAX, IL1A, TP53, TP63, NLRP2, NLRP7, PLCG1, CASP3, CASP5, CASP6, CASP8, GSDMB, GSDMD, PYCARD, BAK1, AIM2, CYCS, and HMGB1) were upregulated in the tumor group. RNA levels of the 29 genes are shown in Figure 2A. To further investigate the interactions of the pyroptosis‐related genes, a PPI analysis was conducted and the results are shown in Figure 2B. The minimum required interaction score was 0.40, and we determined that AIM2, IRF1, PYCARD, IL1B, IL18, NLRP3, HMGB1, CASP8, TNF, IL6, CASP4, NLRC4, CASP5, CASP1, TP53, CYCS, and CASP3 were hub genes. Among them, except for IRF1, IL1B, IL18, CASP1, TNF, CASP4, NLRC4, and CASP1, other genes were all DEGs between normal and tumor tissues. The correlation network is presented in Figure 2C. These findings demonstrated that pyroptosis regulators play a vital role in bladder cancer. The lncRNAs co‐expressed with the 52 pyroptosis regulators were determined by analyzing data of RNA‐seq transcriptome using a co‐expression network (Figure 2D; Table S1).

**FIGURE 3:**
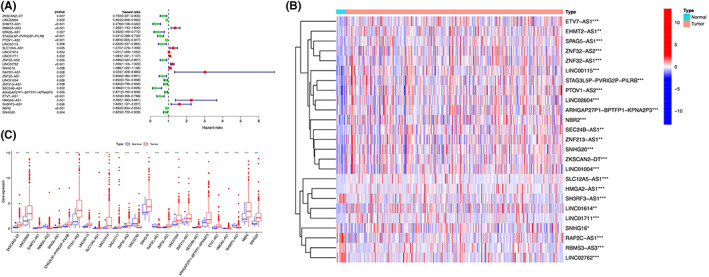
Pyroptosis‐related IncRNAs regulators in bladder cancer. (A) The signature from 25 pyroptosis‐related IncRNAs was screened using univariate Cox regression. (B) The expression of 25 pyroptosis‐related IncRNAs in bladder cancer and normal tissues. *p < 0.05; **p < 0.01; ***p < 0.001. (C) Expression of the pyroptosis‐related IncRNAs. Blue represents normal tissues, and red indicates bladder cancer samples).

Clinical data from TCGA for survival time and survival status were merged with expression of pyroptosis‐related lncRNAs. And 25 lncRNAs related to prognosis were selected using “survival” package with the screening condition “*p* < 0.01” (Table 1). Univariate Cox regression analyses were performed to investigate the relationship between the 25 lncRNAs and overall survival (Figure 3A). Among them, 16 lncRNAs were protective and correlated with a better prognosis. The expression differences of the 25 pyroptosis‐related lncRNAs between bladder cancer and healthy controls were investigated. Results were shown in Figure 3B,C. The expression of the lncRNAs differed significantly between bladder cancer patients and healthy controls. Most of the lncRNAs are highly expressed in tumor group, except for SH3RF3‐AS1, RAP2C‐AS1, RBMS3‐AS3, and LINC02762.

**TABLE 1:** Univariate cox analysis for the top 25 pyroptosis‐related lncRNAs

Gene	HR	Lower limit of 95% CI	Upper limit of 95% CI	*p* Value
ZKSCAN2‐DT	0.699535028528478	0.540752812395627	0.904940750969814	0.00652063683490378
LINC02604	0.903599504951553	0.848174956604762	0.962645806729667	0.00169679492648592
EHMT2‐AS1	0.278015160556069	0.137558402407557	0.56188810095377	0.000362912030342081
RBMS3‐AS3	1.36919971103744	1.142846195435	1.64038508085636	0.000653995362046282
SPAG5‐AS1	0.391679934919641	0.198595002602113	0.772492607611174	0.00683287871766133
STAG3L5P‐PVRIG2P‐PILRB	0.540860727481286	0.397896049520262	0.735192839648158	8.7059190018157e‐05
PTOV1‐AS2	0.88418784032655	0.825416508821544	0.947143809974792	0.000452515421541723
LINC00115	0.621663714392752	0.437198337592662	0.883959842849778	0.00812749066713711
SLC12A5‐AS1	1.26990699848652	1.07569866146788	1.49917801571345	0.00477751148736016
LINC01614	1.03059967802913	1.00939178076677	1.05225316531397	0.00449562910371904
LINC01711	1.08272708131859	1.03077545073946	1.13729710169143	0.00153403315012756
ZNF32‐AS2	0.695796128466417	0.539914430640794	0.896683298155719	0.00506876767208571
LINC02762	1.20880193543091	1.10544338868563	1.32182446795296	3.2085727694881e‐05
SNHG16	1.08794464897492	1.02231187793325	1.1577910662899	0.007930057687796
RAP2C‐AS1	3.0232142193062	1.32791675143149	6.88282921799315	0.00839838370423482
ZNF32‐AS1	0.65613858052125	0.483146150355458	0.891071648882439	0.00696442055997283
LINC01004	0.849513379846671	0.75355974538451	0.957685156298091	0.00765329560130449
ZNF213‐AS1	0.840188319564053	0.738705905447226	0.955612249917633	0.00801912745601882
SEC24B‐AS1	0.266455403337824	0.113401116128042	0.626082744086553	0.00241077153081735
ARHGAP27P1‐BPTFP1‐KPNA2P3	0.871270799363787	0.791894143458059	0.958603889288919	0.0046927464196717
ETV7‐AS1	0.524147621192284	0.358834351025639	0.765619924670759	0.000833546138709331
HMGA2‐AS1	2.26189977874227	1.3897494373656	3.68137627655567	0.00102228254930613
SH3RF3‐AS1	1.60865428809096	1.14672043510695	2.25666914041875	0.00591080351360877
NBR2	0.891825179254477	0.83361178565535	0.954103773529314	0.000886934258366424
SNHG20	0.82318311149968	0.721895426301127	0.938682266668956	0.0036777750562139

Abbreviations: HR, hazard ratio; CI, confidence interval.

### 3.2 Consensus clustering of pyroptosis‐related lncRNAs

**FIGURE 4:**
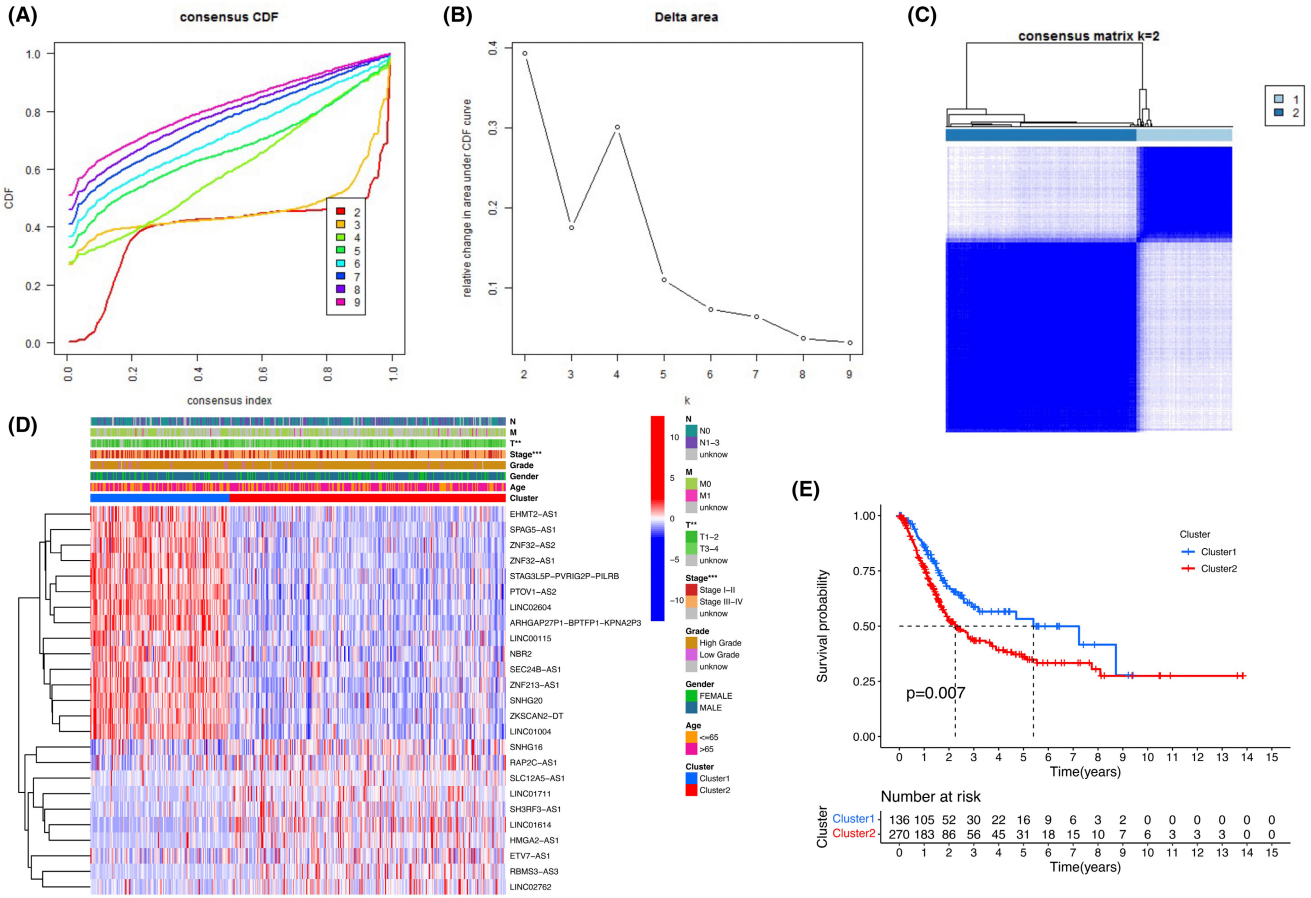
Correlation between the pyroptosis‐related IncRNAs and clinicopathological feature. (A) Model of consensus clustering. (B) Relative change in area under the CDF curve. (C) TCGA‐BLCA cohort was separated into two clusters. (D) The correlation of the two clusters with clinicopathologic features. **p < 0.01; ***p < 0.001. (E) Overall survival of patients in the two clusters.

Consensus clustering was performed, with k = 2–9 in a cumulative distribution function (CDF) (Figure 4A,B), where k represents the cluster count. k = 2 was the optimal clustering parameter (Figure 4C). Survival time and the expression level of the selected lncRNAs were combined. Finally, 406 patients were separated into two clusters, namely, cluster 1 (*n* = 136) and cluster 2 (*n* = 270), depending on expression of the pyroptosis‐related lncRNAs. Of note, early bladder cancer was associated with a cluster 1 regulatory pattern, and advanced stage was mainly associated with the cluster 2 regulatory pattern (*p* < 0.001, Figure 4D). Accordingly, the cluster 1 regulatory pattern has a better survival advantage (Figure 4E).

**FIGURE 5:**
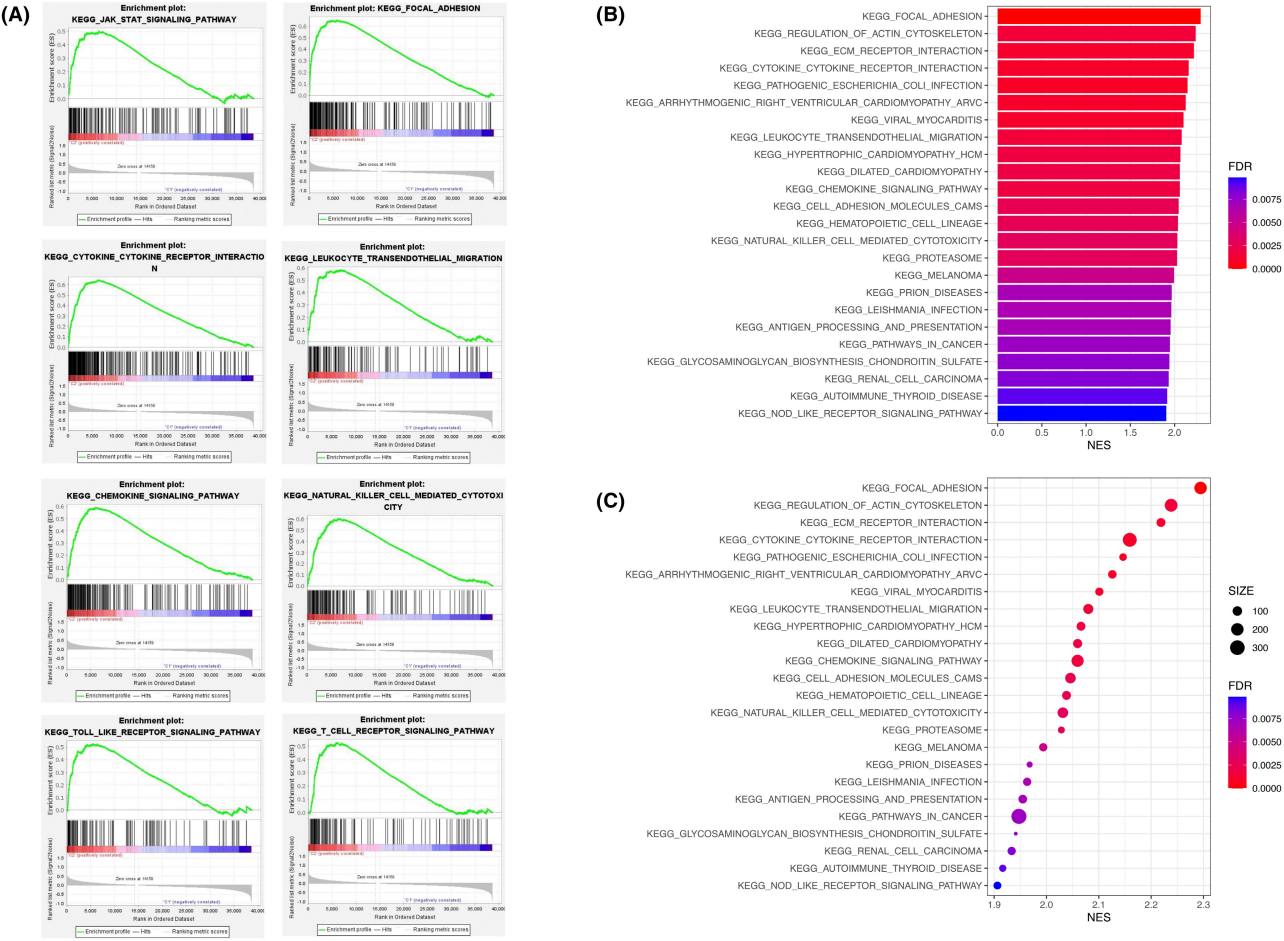
Functional analysis between the two cluster groups. (A) Functions and pathways of the two clusters were analyzed by GSEA. (B) KEGG pathways shown as barplot graph. (C) KEGG enrichment shown as bubble graph.

Active signaling pathways were revealed by GSEA that differed between the two clusters. The filter condition was set as false discovery rate (FDR) *q*‐value <0.05, and the following pathways were found to be active in cluster 2 (Figure 5A): “focal adhesion,” “chemokine signaling pathway”, “leukocyte transendothelial migration”, “cytokine‐cytokine receptor interaction,” “natural killer cell mediated cytotoxicity,” “T cell receptor signaling pathway”, “Toll‐like receptor signaling pathway,” and “JAK/STAT signaling pathway”. These results proved that the clusters 2 is related to immune responses. As for the KEGG pathways barplot and bubble graph (Figure 5B,C), the immune‐associated pathways were highly active in cluster 2. There are no active pathways in cluster 1 using the same filter conditions.

### 3.3 PD‐L1 expression and pyroptosis‐related lncRNAs

**FIGURE 6:**
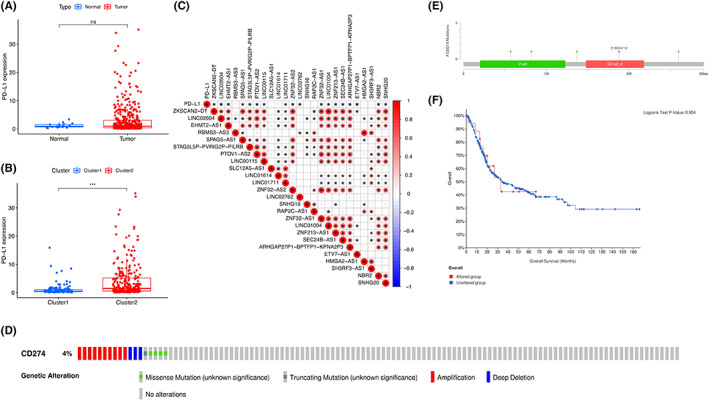
Correlation of PD‐L1 with pyroptosis‐related IncRNAs. (A) The expression level of PD‐L1 in tumor samples and healthy controls in TCGA cohort. (B) PD‐L1 upregulation in cluster 2 subtypes in bladder cancer, ***p < 0.001. (C) Correlation of PD‐L1 and the pyroptosis‐related IncRNAs. (D) PD‐L1 alterations in BLCA cohort. (E) PD‐L1 alterations in BLCA cohort. (F) Overall survival between patients with and without PD‐L1 alterations.

The differences in PD‐L1 expression between tumors and healthy controls and between clusters 1 and 2 were estimated (Figure 6A,B). No significant difference was observed in PD‐L1 expression between normal adjacent tissues and tumor samples. Compared to clusters 1, PD‐L1 expression was upregulated in clusters 2 (*p* < 0.001). PD‐L1 expression had a significantly positive association with the expression levels of LINC01711, LINC02762, and ETV7‐AS, whereas a significantly negative correlation was observed with the expression levels of ZKSCAN2‐DT, LINC02604, EHMT2‐AS1, SPAG5‐AS1, STAG3L5P‐PVRIG2P‐PILRB, PTOV1‐AS2, LINC00115, ZNF32‐AS2, ZNF32‐AS1, LINC01004, ZNF213‐AS1, SEC24B‐AS1, ARHGAP27P1‐BPTFP1‐KPNA2P3, NBR2, and SNHG20 (Figure 3C). In addition, the 25 lncRNAs were positively correlated with each other (Figure 6C).

The types and frequency of PD‐L1 mutations in bladder cancer were determined via cBioPortal. As shown in Oncoprint, PD‐L1 is altered in 4% of bladder cancer patients, including missense mutations, deep deletions, and amplifications (Figure 6D). Most of PD‐L1 alterations are missense mutations. The locations of PD‐L1 mutations in bladder cancer patients were shown in Figure 6E. No statistically significant differences were observed in patients with and without PD‐L1 alterations (Figure 6F).

### 3.4 Consensus clustering for pyroptosis‐related lncRNAs with immune cell

**FIGURE 7:**
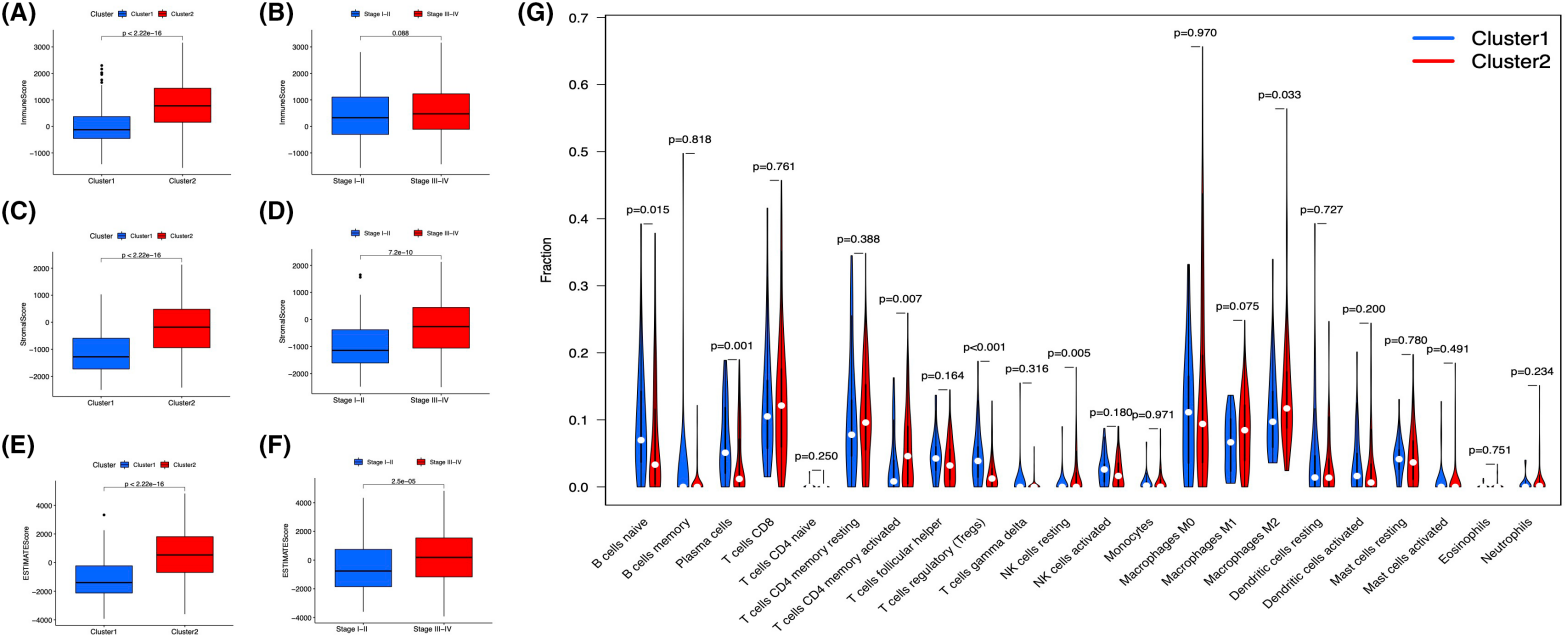
Diverse clinical features and immune cell infiltration in two clusters. The Immunescore (A), Stromalscore (B), and Estimatescore (C) in cluster 1/2 subtypes in TCGA cohort. The Immunescore (D), Stromalscore (E), and Estimatescore (F) in stage I–II/III–IV patients. (G) 22 immune cell types in the TCGA cohort.

Immunescores (Figure 7A) and Stromalscores (Figure 7B) in each sample were calculated, and the two scores were combined to obtain Estimatescore (Figure 7C). There was a significant difference in the Immunescores, Stromalscores, and Estimatescores of the two clusters. The Stromalscores and Estimatescores were positively correlated with the bladder cancer stage (Figure 7D–F), indicating that the purity of tumor cells decreased with cancer progression.

The fractions of 22 immune cell subtypes were analyzed between clusters 1 and 2 (Figure 7G). *p* < 0.05 was used as the screening condition. Cluster 1 had higher infiltration of naïve B cells, plasma cells, and regulatory T cells (Tregs), whereas cluster 2 was associated with memory‐activated T cells CD4, resting NK cells, and M2 macrophages.

### 3.5 Construction and validation of prognostic signatures

**FIGURE 8:**
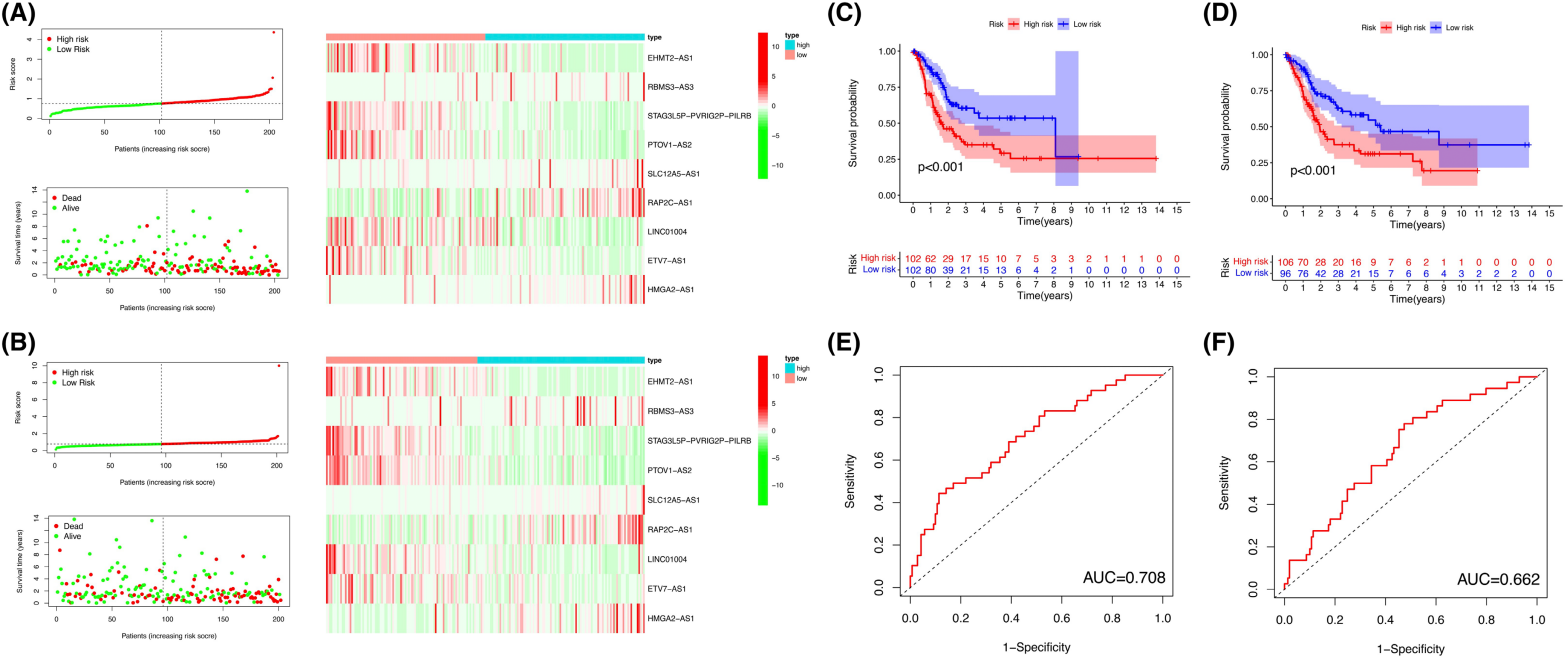
Construction of prognostic signatures. Risk score, overall survival, and survival status in the training (A) and testing cohort (B) Kaplan–Meier curves of overall survival in the training (C) and testing cohort (D) ROC curves in the training (E) and testing cohort (F).

The usefulness of pyroptosis‐related lncRNAs for predicting bladder cancer patient prognosis was evaluated. And 406 patients separated into the training cohort (204 patients) and testing cohort (202 patients). A LASSO regression analysis was performed based on the expression levels of the 25 pyroptosis‐related lncRNAs in the TCGA training cohort. From this, nine important pyroptosis‐related lncRNAs were identified, which are EHMT2‐AS1, RBMS3‐AS3, STAG3L5P‐PVRIG2P‐PILRB, PTOV1‐AS2, SLC12A5‐AS1, RAP2C‐AS1, LINC01004, ETV7‐AS1, and HMGA2‐AS1 (Table 2). Patients were classified into high‐ and low‐risk groups based on the median risk scores estimated using the coefficients from the LASSO algorithm. The relationships between expression signatures of nine pyroptosis‐related lncRNAs, risk score, overall survival, and survival status were shown (Figure 8A,B). The results indicate that among the nine lncRNAs, five selected lncRNAs are highly expressed in the low‐risk group (EHMT2‐AS1, STAG3L5P‐PVRIG2P‐PILRB, PTOV1‐AS2, LINC01004 and ETV7‐AS1).

**TABLE 2:** Pyroptosis‐related lncRNAs coefficient

Gene	Coefficient
EHMT2‐AS1	−0.28195
RBMS3‐AS3	0.030943
STAG3L5P‐PVRIG2P‐PILRB	−0.16981
PTOV1‐AS2	−0.03363
SLC12A5‐AS1	0.330934
RAP2C‐AS1	0.65514
LINC01004	−0.00434
ETV7‐AS1	−0.29828
HMGA2‐AS1	0.32351

Overall survival between the two groups were further analyzed (Figure 8C and D). Overall survival was significantly longer in the low‐risk group, irrespective of training or testing group (*p* < 0.001). ROC curve was generated, and AUC values were 0.708 and 0.662 in training and testing groups, respectively (Figure 8E,F).

### 3.6 Correlation of risk score with clinicopathological factors, clusters, and immune‐scores

**FIGURE 9:**
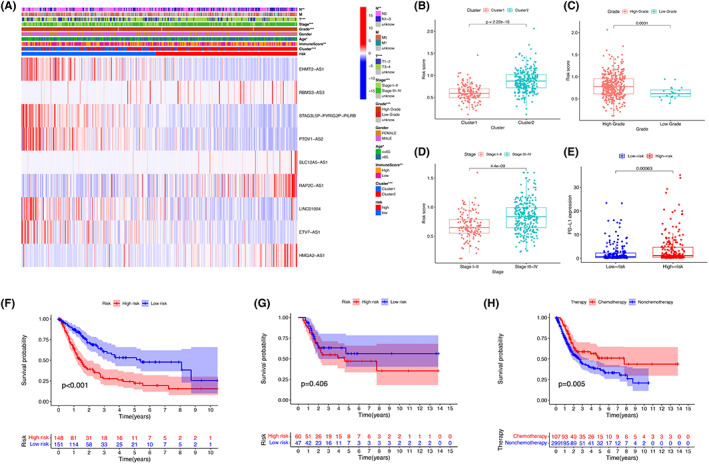
Prognostic risk scores correlated with clinicopathological features and immunoscore. (A) Clinicopathologic features of high‐ and low‐risk groups were shown in heatmap. *p < 0.05; **p < 0.01; ***p < 0.001. Distribution of risk scores stratified by grade (B), stage (C), and cluster 1/2 (D). (E) PD‐L1 expression level in training cohort. (F) Kaplan–Meier curves for the overall survival of high‐ and low‐ risk patients receiving (F) and not receiving chemotherapy (G). (H) Kaplan–Meier curves for the overall survival of patients receiving and not receiving chemotherapy.

Clinicopathological factors, cluster analysis and the immune‐scores were compared in high‐ and low‐risk groups. Expression differences of the 9 selected pyroptosis‐related lncRNAs were visualized (Figure 9A). Absolute expression of the 5 pyroptosis‐related lncRNAs was lower in the high‐group, namely EHMT2‐AS1, STAG3L5P‐PVRIG2P‐PILRB, PTOV1‐AS2, RAP2C‐AS1, and ETV7‐AS1. Higher risk scores were observed in high grade (Figure 9B), stage III–IV (Figure 9C) and cluster 2 (Figure 9D). PD‐L1 expression and risk score were further evaluated and a significant correlation was found between the high‐ and low‐risk groups (Figure 9E). The prognostic role of pyroptosis‐related lncRNAs in BLCA patients receiving chemotherapy was also analyzed. Patients with high‐risk score had worse prognosis when receiving chemotherapy (Figure 9F). There was no significant difference in prognosis between patients without receiving chemotherapy (Figure 9G). When compared with patients receiving chemotherapy, those who did not receive chemotherapy had worse prognosis (Figure 9H).

The differences in overall survival of among gender, age, stage, grade, TNM staging were also determined. Except in stage I‐II and the low‐grade group, all the rest subgroups had a higher overall survival in low‐risk groups (Figure S1).

**FIGURE 10:**
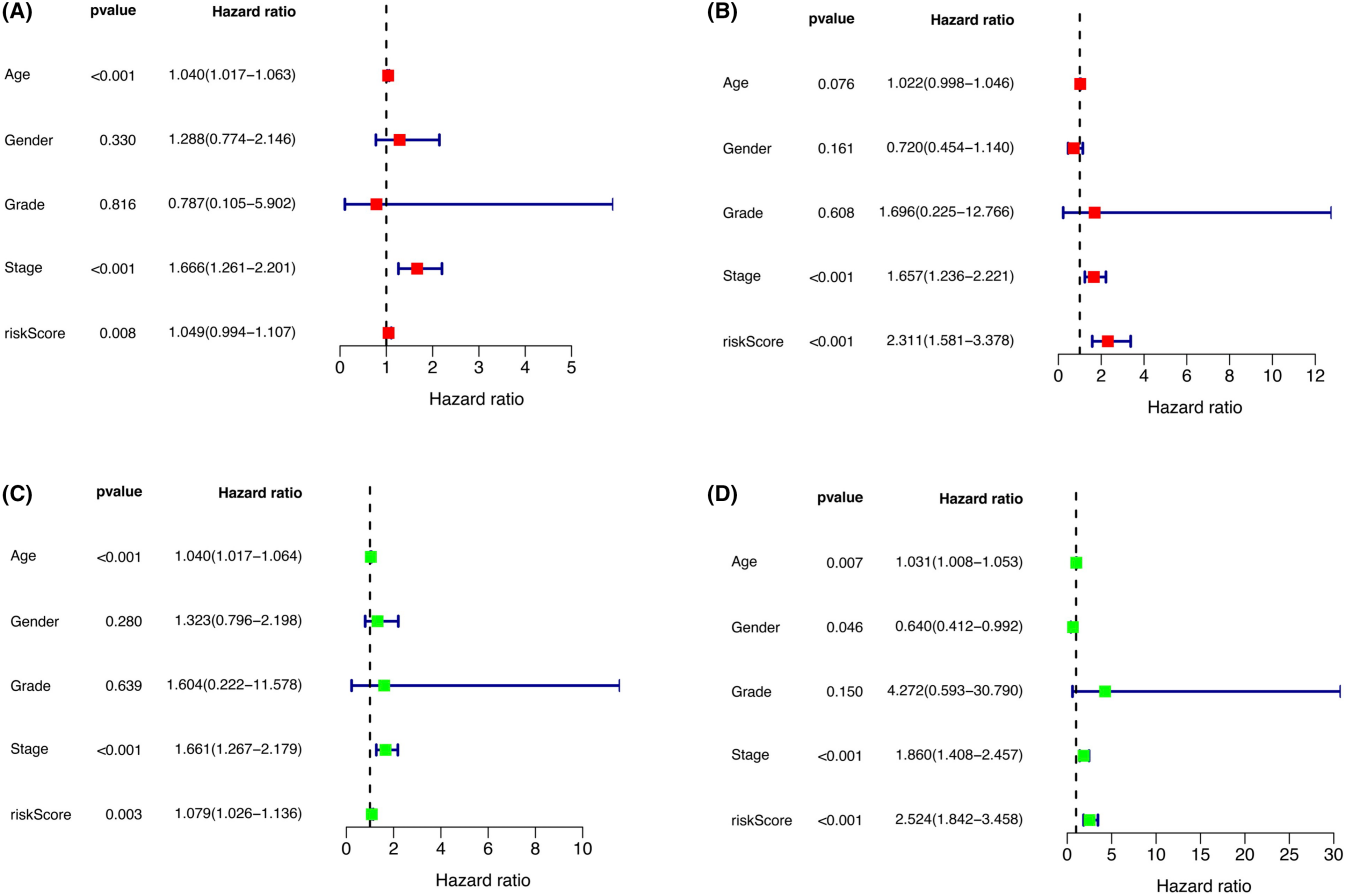
Cox regression analysis in the training and testing group. Univariate Cox regression in the training (A) and testing cohort (B) Multiple Cox regression in the training (C) and testing cohort (D).

Univariate and multivariate Cox analyses for overall survival were performed (Figure 10A–D). Multivariate analysis indicated that stage, age, and risk score were independent factors for worse prognosis.

### 3.7 Correlation of pyroptosis‐related lncRNAs with immunocytes

**FIGURE 11:**
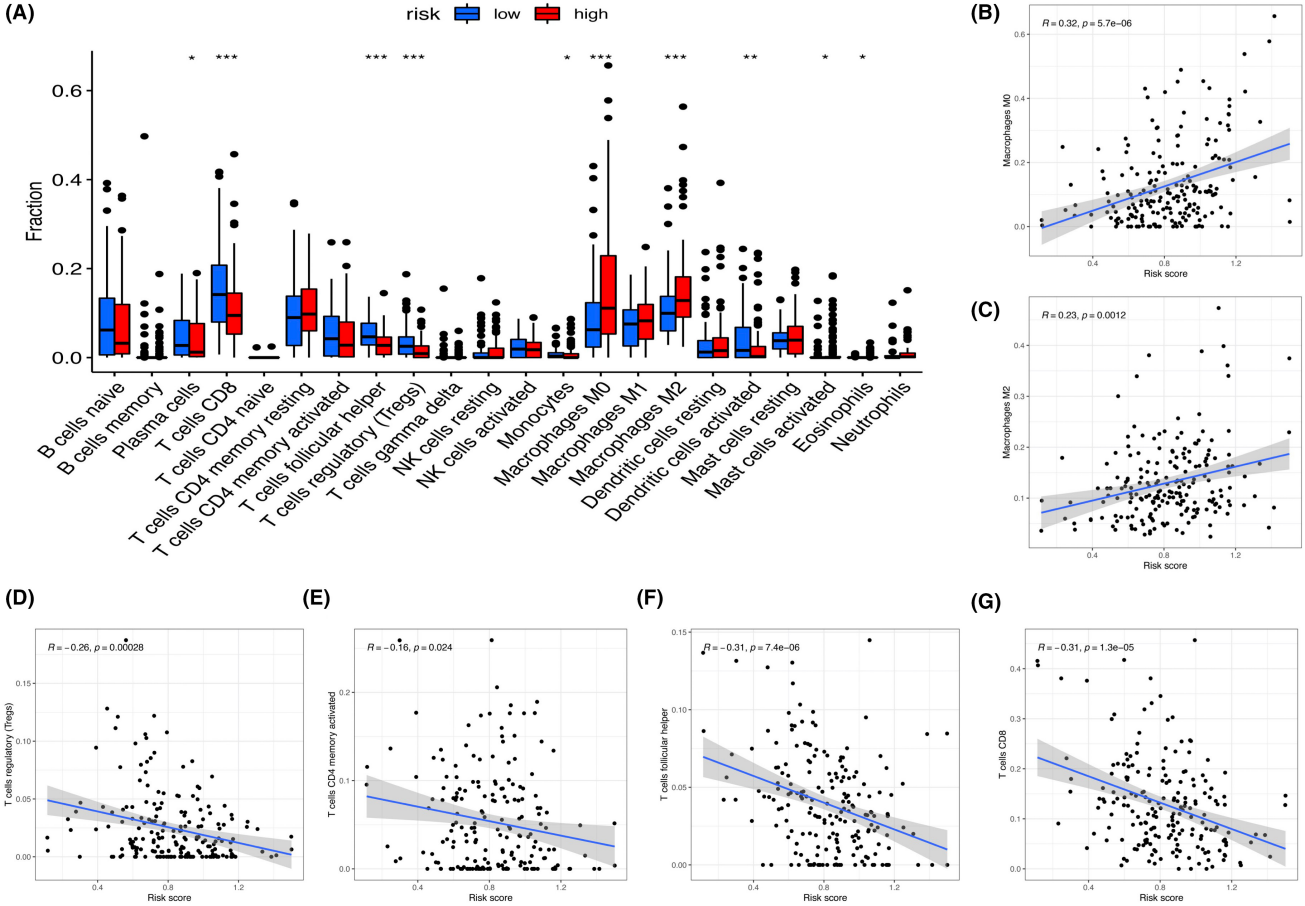
Correlation of the risk score and infiltration of immune cells. (A) The relationship between risk score and infiltration levels of 22 immune cell types. M0 macrophages (B); M2 macrophages (C); regulatory T cells (Tregs) (D); memory‐activated CD4 T cells (E); follicular helper T cells (F); and CD8 T cells (G).

Risk scores with the immune cell infiltration of 22 subtypes were correlated (Figure 11A). Risk score was positively correlated with M0 macrophages (*p* = 5.7e−06) (Figure 11B) and M2 macrophages (*p* = 0.0012) (Figure 11C), and negatively correlated with regulatory T cells (Tregs) (*p* = 0.00028) (Figure 11D), memory activated CD4+ T cells (*p* = 0.024) (Figure 11E), follicular helper T cells (*p* = 7.4e−06) (Figure 11F), and CD8+ T cells (*p* = 1.3e−05) (Figure 11G). The finding suggested that the pyroptosis‐related lncRNA risk signature is related in the immune microenvironment of BLCA.

### 3.8 Correlation of pyroptosis‐related lncRNAs‐associated prognostic model with TMB

**FIGURE 12:**
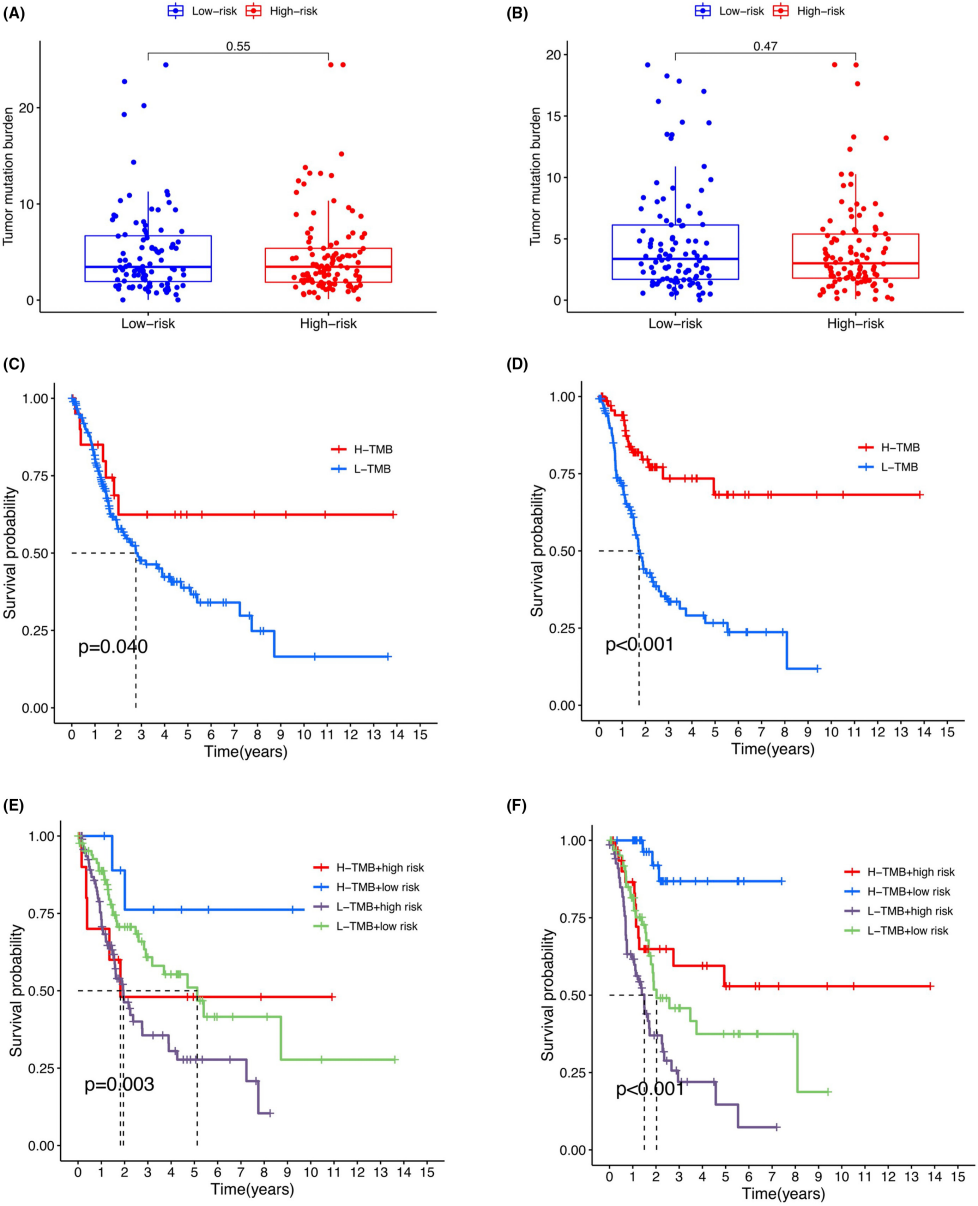
Relationship of the PLPM‐based risk signature with TMB. Comparison of TMB between PLPM‐high and PLPM‐low groups in training (A) and testing cohort (B) Kaplan–Meier survival analysis based on the TMB in training (C) and testing cohort (D) Kaplan–Meier survival analysis for four groups in training (E) and testing cohort (F).

There were no significant differences in TMB between patients with high and low PLPM in the training or testing group (Figure 12A,B). However, high TMB was associated with better overall survival (Log‐rank test, *p* < 0.001, *p* = 0.040, respectively, Figure 12C,D). We investigated whether the combination of PLPM and TMB could be a better biomarker for prognosis. PLPM and TMB were integrated to stratify all the samples into: TMB^high^/PLPM^low^, TMB^low^/PLPM^low^, TMB^high^/PLPM^high^, and TMB^low^/PLPM^high^ groups. As shown in Figure 12E,F, there were significant differences among all groups (Log‐rank test, *p* < 0.001, *p* = 0.003, respectively), and patients in the TMB^high^/PLPM^low^ group exhibited the highest overall survival.

### 3.9 Establishment and validation of a nomogram

**FIGURE 13:**
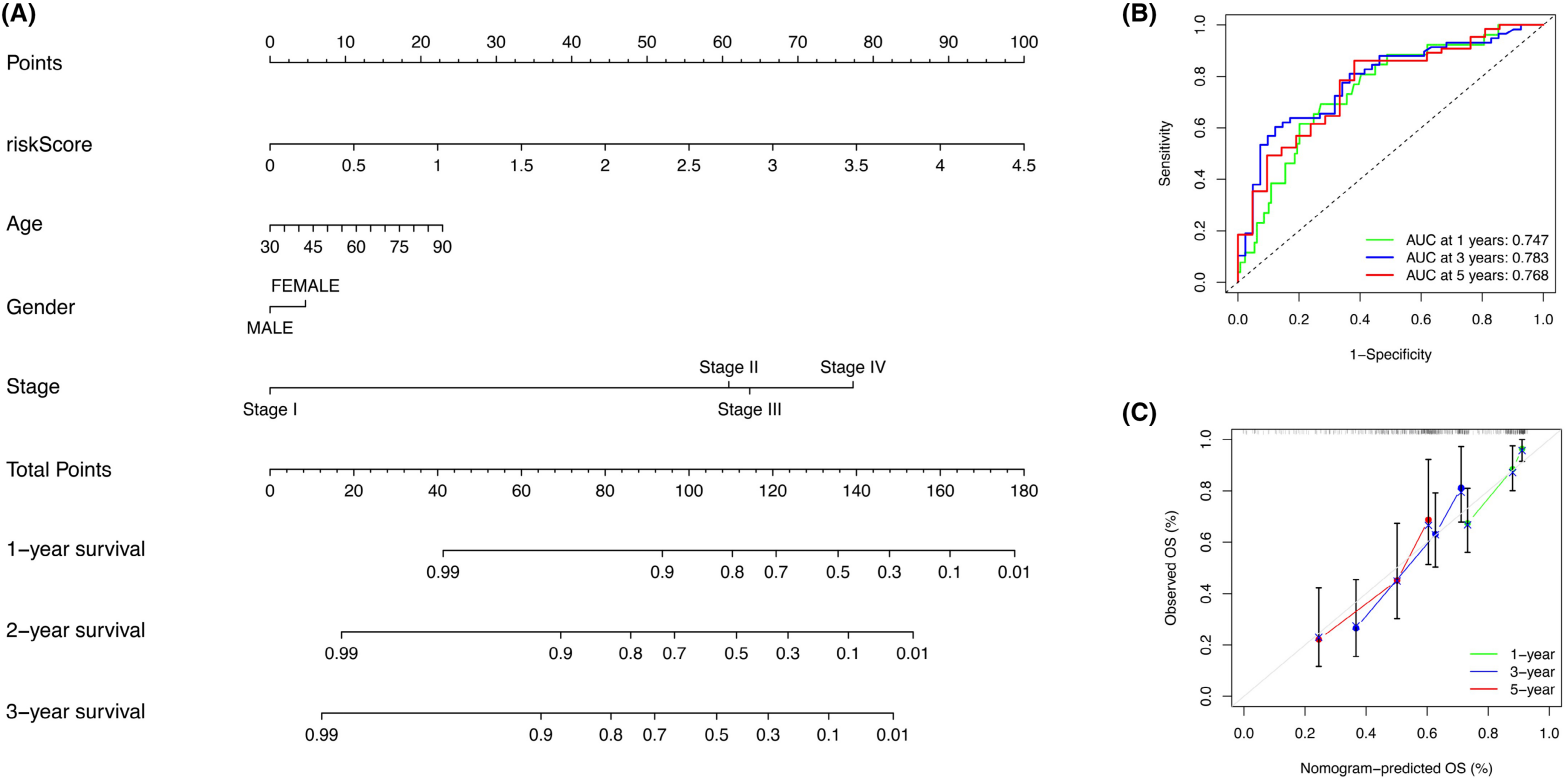
Construction and validation of the nomogram. (A) Nomogram constructed with clinical characterization and pyroptosis‐related lncRNAs risk signature. (B) ROC curve and AUC for 1‐, 3‐, and 5‐ year overall survival. (C) The calibration plot of the nomogram.

Based on the clinical factors and risk score, a nomogram was constructed (Figure 13A). The prediction value of the nomogram was determined by calibration curve and the actual survival outcomes were shown from the 45‐degree line. The AUCs for the 1‐, 3‐, and 5‐year were 0.747, 0.783, and 0.768, respectively (Figure 13B). The nomogram had similar performance to that of an ideal model (Figure 13C). These findings suggested that the nomogram combining the signature and clinical factors had optimal prognostic accuracy.

## 4 DISCUSSION

Pyroptosis is a distinct type of programmed cell death, which is characterized by DNA fragmentation, chromatin condensation, and leakage of cell content.^33^ Pyroptosis can be chemically induced and may affect all stages of carcinogenesis. As an inflammatory form of cell death, pyroptosis would active the immune system.^34^ Pyroptosis is a promising new target in cancer treatment, while many issues remain unsolved such as the interconnection between pyroptosis and host immunogenicity.

LncRNAs serve essential roles during tumorigenesis. With advanced sequencing methods, a growing number of lncRNAs have been identified in various cancer types.^35^ LncRNAs appear to regulate biological behaviors through epigenetic, transcription and post‐transcriptional processing. Increasing evidences suggest that lncRNAs are vital in mediating pyroptosis. Up to now, there are no studies on the prognostic role of pyroptosis‐associated lncRNAs in bladder cancer.

We first investigated pyroptosis‐related lncRNAs signature of bladder cancer based on the TCGA dataset. Our analyses uncovered 29 pyroptosis‐related DEGs. GSEA and KEGG analyses indicated the genes are involved in extracellular matrix (ECM) receptor interaction, regulation of actin cytoskeleton, focal adhesion, cytokine receptor interaction, Toll‐like receptor signaling pathway, natural killer cell mediated cytotoxicity, JAK/STAT signaling pathway, and T cell receptor signaling pathways.

Overall, nine differently expressed pyroptosis‐related lncRNAs were determined to be independent factors for the prognosis of bladder cancer. Among the identified lncRNAs, four of them were associated with tumor progression, namely, RBMS3‐AS3, SLC12A5‐AS1, RAP2C‐AS1, and HMGA2‐AS1. However, the role of the lncRNAs have conflicting results in other studies. For example, RBMS3‐AS3 was reported to act as a microRNA‐4534 sponge to upregulate VASH1 and inhibit prostate cancer.^36^ SLC12A5‐AS1 was one of the top 25 upregulated lncRNAs in head and neck squamous cell carcinoma.^37^ Low expression of RAP2C‐AS1 was associated with lymphatic invasion in clear cell carcinoma.^38^ HMGA2‐AS1 was reported to be involved in pancreatic cancer progression.^39^ On the other hand, five protective lncRNAs are identified based on the results of our study. EHMT2‐AS1 is lower in the high‐risk versus low‐risk group in bladder cancer, and is one of the m6A‐related lncRNAs for prognosis.^40^ STAG3L5P‐PVRIG2P‐PILRB and other lncRNAs comprise a prognostic signature for survival of patients of bladder cancer.^41^ PTOV1‐AS2 is one of the five m6A‐related lncRNAs of risk score signature of pancreatic cancer.^42^ However, the prognostic value in cancer and pyroptosis are lacking for 2 lncRNAs (LINC01004 and ETV7‐AS1). These results suggested that the role of pyroptosis in tumor cell growth are promoting or inhibiting in different cancer types. The therapeutic directions of lncRNAs for the treatment of bladder cancer deserve further study.

Pyroptotic cells release cellular components, which induce lymphocyte infiltration and inflammatory responses. Tumor‐infiltrating lymphocytes induce pyroptosis of tumor cells, which is a positive‐feedback loop of anti‐tumor immunity.^14^ Pyroptosis in target cells could sensitize ICI‐resistant cancers to checkpoint blockade.^43^ In this study, bladder patients were stratified into two categories of high‐ and low‐risk based on this prognostic model. The roles of immune infiltrating cells in tumor microenvironment and in the prognosis of bladder cancer were then explored. The results show that CD8+ T cells and activated dendritic cells of the high‐risk group were significantly reduced compared with those in low‐risk group, whereas the immune cells promoting tumor proliferation such as M2 macrophages were increased. The results suggested that pyroptosis is correlated with a proportion of immune cells in bladder cancer.

Our results indicated PD‐L1 expression is upregulated in cluster 2 compared with cluster 1 subtype. To date, most research on PD‐L1 has focused on its immune checkpoint function. A non‐immune checkpoint function of PD‐L1 was reported, which is involved in the pyroptosis pathway. PD‐L1‐mediated expression of gasdermin C (GSDMC) could switch cancer apoptosis to pyroptosis.^44^ In human pulmonary arterial smooth muscle cells, PD‐L1 is required for hypoxia‐induced pyroptosis, indicating that PD‐L1‐mediated pyroptosis also exists in other types of cells.^45^

A previous study investigated the correlation of genes involved in pyroptosis and TMB in pan‐cancer.^12^ In bladder cancer patients, the correlation is not significant. We analyzed the TMB status for high‐ and low‐risk bladder cancer patients. Although the TMB status are not significantly different in the two groups, TMB alone is a prognostic marker in bladder cancer patients. Furthermore, when TMB and risk score were combined together, they can jointly stratify bladder cancer patients into groups with different prognosis.

In our study, patients receiving chemotherapy with high‐risk scores had a worse prognosis, while there was no survival difference of high‐ and low‐ risk score in patients not receiving chemotherapy. Recently findings have also revealed gasdermin E (GSDME) enhances cisplatin sensitivity by mediating pyroptosis to trigger immunocyte infiltration in NSLCC.^46^ Further studies are warranted to discover the effect of chemotherapy on pyroptosis inducers in bladder cancer. However, data from TCGA are not sufficient to analysis the pyroptosis‐related lncRNAs signature based on immune checkpoint inhibitors.

Our study has limitations. Firstly, clinical and sequencing data are derived from the TCGA database, therefore, the prognostic model require further validation with real‐world data. Secondly, the exploration of pyroptosis‐related lncRNAs signature and tumor immune microenvironment is preliminary. Thirdly, well‐known prognostic factors such as treatment with immune checkpoint inhibitors and other tumor markers were not incorporated into the nomogram as information on these parameters were incomplete.

Our study explored pyroptosis‐related lncRNAs as a prognosis signature of bladder cancer, which can inform future treatment options for the diseases. Findings of our research shed light on potential biomarker and therapeutic targets in pyroptosis signaling pathways.

## AUTHOR CONTRIBUTIONS

Zhiqiang Wang, Zhentao Yu and Ling Peng designed and supervised the study. Peng Wang, Zhiqiang Wang, Liping Zhu and Ling Peng analyzed the data and wrote the original draft. Ling Peng, Zhentao Yu, Yilan Sun, Leandro Castellano and Justin Stebbing edited the draft. All the authors have read and approved the final manuscript.

## FUNDING INFORMATION

This study was supported by a grant from Medical Science Research Foundation of Health Bureau of Zhejiang Province (Grant number: 2022KY545) and a grant from the Administration of Traditional Chinese Medicine of Zhejiang Province (Grant number: 2022ZA021).

## CONFLICT OF INTEREST

JS' conflicts can be found at https://www.nature.com/onc/editors. None are relevant here. Other authors none declared.

## ETHICS APPROVAL

None applicable.

## CONSENT FOR PUBLICATION

None applicable.

## Supporting information


Figure S1

Table S1

## Data Availability

Publicly available datasets were analyzed in this study.
