# Supplementary figures and images for: A pyroptosis‐related lncRNA signature in bladder cancer
Source: Cancer Med. 2022 Oct 13;12(5):6348–64. doi: 10.1002/cam4.5344 (PMC10028168; doi:10.1002/cam4.5344)

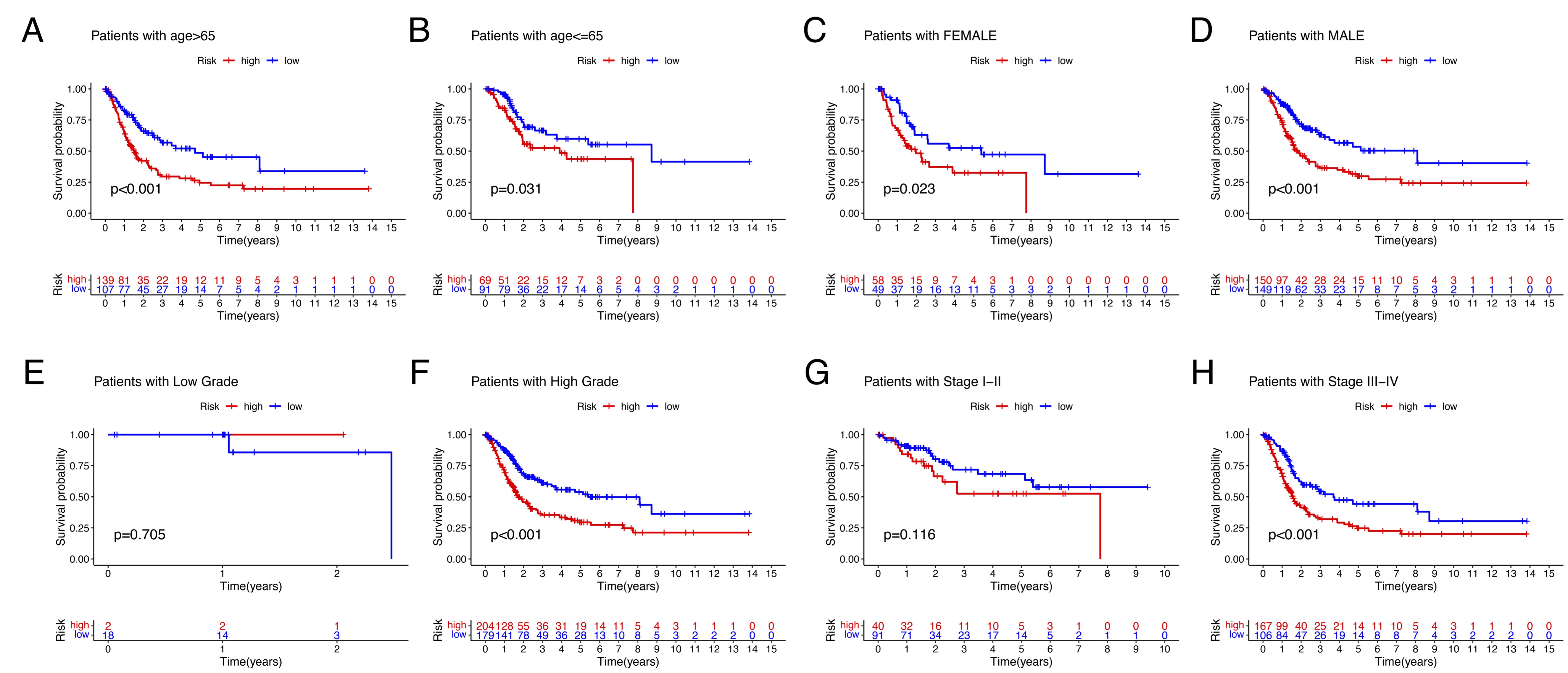

Supplement: Supplementary file 1 — Figure S1 [file CAM4-12-6348-s002.jpg]
