# Supplementary material for: A pyroptosis‐related lncRNA signature in bladder cancer
Source: Cancer Med. 2022 Oct 13;12(5):6348–64. doi: 10.1002/cam4.5344 (PMC10028168; doi:10.1002/cam4.5344)
Supplement: Supplementary file 2 — Table S1 [file CAM4-12-6348-s001.doc]

# Supplementary Table.

## Supplementary Table 1. LncRNAs and pyroptosis co-expression network.

| Pyroptosis | lncRNA | cor | P value | Regulation |
| --- | --- | --- | --- | --- |
| CHMP4A | LINC01176 | 0.42618572 | 1.06E-19 | Positive |
| NLRC4 | ENTPD1-AS1 | 0.44116906 | 3.81E-21 | Positive |
| SCAF11 | ENTPD1-AS1 | 0.539568547 | 1.19E-32 | Positive |
| GZMB | MAFA-AS1 | 0.604363025 | 1.40E-42 | Positive |
| ELANE | IL6-AS1 | 0.412428472 | 1.97E-18 | Positive |
| IL6 | IL6-AS1 | 0.593120242 | 1.08E-40 | Positive |
| NLRP3 | IL6-AS1 | 0.464025579 | 1.70E-23 | Positive |
| SCAF11 | LAMC1-AS1 | 0.441521508 | 3.51E-21 | Positive |
| SCAF11 | DTX2P1-UPK3BP1-PMS2P11 | 0.41643811 | 8.52E-19 | Positive |
| SCAF11 | C21orf62-AS1 | 0.46025482 | 4.27E-23 | Positive |
| CHMP4A | IDH1-AS1 | 0.431910518 | 3.04E-20 | Positive |
| CHMP4A | ZKSCAN2-DT | 0.420547753 | 3.57E-19 | Positive |
| GSDMB | ZKSCAN2-DT | 0.407430512 | 5.49E-18 | Positive |
| PJVK | ZKSCAN2-DT | 0.472669927 | 1.97E-24 | Positive |
| GSDMB | LINC02604 | 0.431513087 | 3.32E-20 | Positive |
| PJVK | LINC02604 | 0.421697935 | 2.80E-19 | Positive |
| TIRAP | NDUFA6-DT | 0.458645224 | 6.31E-23 | Positive |
| NOD2 | TM4SF19-AS1 | 0.423620314 | 1.85E-19 | Positive |
| CHMP4A | SNHG11 | 0.443646884 | 2.16E-21 | Positive |
| NLRP6 | LINC00861 | 0.423874525 | 1.75E-19 | Positive |
| NLRC4 | LINC02285 | 0.666901829 | 1.36E-54 | Positive |
| NLRP3 | LINC02285 | 0.508646934 | 1.26E-28 | Positive |
| CHMP2A | RAB11B-AS1 | 0.455530351 | 1.33E-22 | Positive |
| PJVK | IQCH-AS1 | 0.433437605 | 2.17E-20 | Positive |
| SCAF11 | STX18-AS1 | 0.443093416 | 2.45E-21 | Positive |
| PJVK | INTS6-AS1 | 0.453042087 | 2.41E-22 | Positive |
| SCAF11 | MANEA-DT | 0.401010434 | 2.00E-17 | Positive |
| GSDMC | SH3PXD2A-AS1 | 0.451747953 | 3.27E-22 | Positive |
| PJVK | LINC01089 | 0.488795655 | 2.99E-26 | Positive |
| NOD2 | LINC01615 | 0.427819948 | 7.46E-20 | Positive |
| SCAF11 | KLF7-IT1 | 0.486066095 | 6.18E-26 | Positive |
| SCAF11 | LINC00649 | 0.448852403 | 6.46E-22 | Positive |
| PJVK | IGBP1-AS1 | 0.430731126 | 3.94E-20 | Positive |
| CHMP2A | SPINT1-AS1 | 0.481784758 | 1.90E-25 | Positive |
| CASP1 | LINC02100 | 0.415965429 | 9.41E-19 | Positive |
| CASP5 | LINC02100 | 0.446599767 | 1.09E-21 | Positive |
| IRF1 | LINC02100 | 0.45585112 | 1.23E-22 | Positive |
| GSDMB | EHMT2-AS1 | 0.498171302 | 2.36E-27 | Positive |
| SCAF11 | FAM13A-AS1 | 0.463361098 | 2.00E-23 | Positive |
| NLRC4 | RBMS3-AS3 | 0.504192494 | 4.44E-28 | Positive |
| SCAF11 | TBL1XR1-AS1 | 0.512502866 | 4.18E-29 | Positive |
| IL1B | LINC01419 | 0.443437324 | 2.27E-21 | Positive |
| SCAF11 | GAS8-AS1 | 0.435255553 | 1.45E-20 | Positive |
| SCAF11 | RC3H1-IT1 | 0.447358561 | 9.15E-22 | Positive |
| PLCG1 | CEP83-DT | 0.424498589 | 1.53E-19 | Positive |
| HMGB1 | TMPO-AS1 | 0.417860529 | 6.32E-19 | Positive |
| SCAF11 | MORC2-AS1 | 0.440908991 | 4.04E-21 | Positive |
| NOD1 | GARS1-DT | 0.535087551 | 4.84E-32 | Positive |
| SCAF11 | GARS1-DT | 0.442949646 | 2.53E-21 | Positive |
| SCAF11 | SPAG5-AS1 | 0.495783355 | 4.55E-27 | Positive |
| SCAF11 | UBL7-AS1 | 0.465541199 | 1.17E-23 | Positive |
| PJVK | STAG3L5P-PVRIG2P-PILRB | 0.456753644 | 9.94E-23 | Positive |
| GZMB | FAM225A | 0.654816855 | 4.76E-52 | Positive |
| SCAF11 | MAL2-AS1 | 0.465853668 | 1.08E-23 | Positive |
| SCAF11 | TXNDC12-AS1 | 0.512303646 | 4.43E-29 | Positive |
| NLRC4 | LINC01914 | 0.419215249 | 4.74E-19 | Positive |
| PJVK | PTOV1-AS2 | 0.462144098 | 2.70E-23 | Positive |
| SCAF11 | PAXIP1-AS2 | 0.470806096 | 3.15E-24 | Positive |
| TIRAP | PAXIP1-AS2 | 0.4773158 | 6.04E-25 | Positive |
| GZMB | PRDM16-DT | 0.51237207 | 4.34E-29 | Positive |
| SCAF11 | LIPC-AS1 | 0.427775718 | 7.53E-20 | Positive |
| SCAF11 | ANKRD10-IT1 | 0.453511909 | 2.16E-22 | Positive |
| TIRAP | ANKRD10-IT1 | 0.41705441 | 7.48E-19 | Positive |
| GSDMB | CTBP1-AS | 0.41620994 | 8.94E-19 | Positive |
| SCAF11 | NCBP2-AS1 | 0.557561532 | 3.43E-35 | Positive |
| TP63 | MIR4713HG | 0.425075617 | 1.35E-19 | Positive |
| SCAF11 | MIR4713HG | 0.417633222 | 6.63E-19 | Positive |
| SCAF11 | FMR1-IT1 | 0.508396847 | 1.35E-28 | Positive |
| SCAF11 | DLEU1 | 0.517333023 | 1.03E-29 | Positive |
| CHMP4A | VASH1-AS1 | 0.463520921 | 1.93E-23 | Positive |
| ELANE | LINC01082 | 0.574654765 | 9.41E-38 | Positive |
| SCAF11 | NUTM2B-AS1 | 0.474534767 | 1.23E-24 | Positive |
| SCAF11 | LINC01675 | 0.410748016 | 2.78E-18 | Positive |
| NLRC4 | STARD13-AS | 0.402845585 | 1.39E-17 | Positive |
| SCAF11 | RNF216-IT1 | 0.463415746 | 1.98E-23 | Positive |
| NLRP6 | LINC00426 | 0.450492801 | 4.40E-22 | Positive |
| GSDMB | LINC00894 | 0.444433741 | 1.80E-21 | Positive |
| SCAF11 | WASHC5-AS1 | 0.554221666 | 1.04E-34 | Positive |
| SCAF11 | SGMS1-AS1 | 0.466300944 | 9.70E-24 | Positive |
| SCAF11 | NR2F2-AS1 | 0.444859552 | 1.63E-21 | Positive |
| PJVK | LINC00115 | 0.404005943 | 1.10E-17 | Positive |
| SCAF11 | OGFRP1 | 0.400115107 | 2.39E-17 | Positive |
| SCAF11 | LINC01409 | 0.522434837 | 2.28E-30 | Positive |
| GZMA | SLC12A5-AS1 | 0.908803274 | 1.69E-158 | Positive |
| CHMP4A | TMED2-DT | 0.428389773 | 6.59E-20 | Positive |
| GSDMB | GEMIN7-AS1 | 0.413543655 | 1.56E-18 | Positive |
| PJVK | GEMIN7-AS1 | 0.473270401 | 1.69E-24 | Positive |
| SCAF11 | SSBP3-AS1 | 0.501904496 | 8.42E-28 | Positive |
| GSDMC | SCAT1 | 0.431986707 | 2.99E-20 | Positive |
| GSDMB | CAPN10-DT | 0.414038901 | 1.41E-18 | Positive |
| PJVK | CAPN10-DT | 0.433030675 | 2.37E-20 | Positive |
| SCAF11 | LINC02163 | 0.467850976 | 6.60E-24 | Positive |
| SCAF11 | LINC02109 | 0.409210771 | 3.81E-18 | Positive |
| ELANE | MIR223HG | 0.409855498 | 3.34E-18 | Positive |
| NLRC4 | MIR223HG | 0.482489765 | 1.58E-25 | Positive |
| NLRP3 | MIR223HG | 0.430277865 | 4.36E-20 | Positive |
| CHMP4A | LINC02413 | 0.422769185 | 2.22E-19 | Positive |
| ELANE | PCAT19 | 0.454069611 | 1.89E-22 | Positive |
| IL6 | PCAT19 | 0.468157475 | 6.12E-24 | Positive |
| SCAF11 | GRHL3-AS1 | 0.439787203 | 5.21E-21 | Positive |
| TIRAP | GRHL3-AS1 | 0.436076251 | 1.20E-20 | Positive |
| ELANE | LINC01081 | 0.500166344 | 1.36E-27 | Positive |
| SCAF11 | LAMTOR5-AS1 | 0.520872145 | 3.62E-30 | Positive |
| GSDMB | MCCC1-AS1 | 0.431057819 | 3.67E-20 | Positive |
| SCAF11 | C1orf220 | 0.40731758 | 5.61E-18 | Positive |
| TIRAP | C1orf220 | 0.430329201 | 4.31E-20 | Positive |
| SCAF11 | UBE2Q1-AS1 | 0.472161175 | 2.24E-24 | Positive |
| SCAF11 | YEATS2-AS1 | 0.476857113 | 6.79E-25 | Positive |
| SCAF11 | MIR2052HG | 0.478343249 | 4.63E-25 | Positive |
| SCAF11 | LINC02466 | 0.402819076 | 1.39E-17 | Positive |
| ELANE | LINC02489 | 0.507856593 | 1.58E-28 | Positive |
| GZMA | LINC01397 | 0.697406425 | 1.39E-61 | Positive |
| SCAF11 | SNHG4 | 0.460671746 | 3.86E-23 | Positive |
| SCAF11 | GTF3C2-AS1 | 0.546254689 | 1.41E-33 | Positive |
| SCAF11 | NARF-IT1 | 0.450977027 | 3.92E-22 | Positive |
| CHMP2A | CD2BP2-DT | 0.401165554 | 1.94E-17 | Positive |
| SCAF11 | FTX | 0.528471224 | 3.70E-31 | Positive |
| GZMB | APCDD1L-DT | 0.532424741 | 1.10E-31 | Positive |
| NLRC4 | APCDD1L-DT | 0.401236224 | 1.91E-17 | Positive |
| NLRC4 | LINC01775 | 0.521421206 | 3.08E-30 | Positive |
| NLRC4 | WWTR1-IT1 | 0.474518098 | 1.23E-24 | Positive |
| SCAF11 | ARAP1-AS2 | 0.479771022 | 3.21E-25 | Positive |
| ELANE | LINC01781 | 0.409248759 | 3.78E-18 | Positive |
| NLRP6 | LINC01781 | 0.405075385 | 8.85E-18 | Positive |
| BAX | CYTOR | 0.40230833 | 1.54E-17 | Positive |
| GZMB | CYTOR | 0.751292615 | 2.30E-76 | Positive |
| NOD1 | LINC02804 | 0.467486728 | 7.23E-24 | Positive |
| NLRC4 | LRRC8C-DT | 0.542754768 | 4.34E-33 | Positive |
| NLRC4 | GNG12-AS1 | 0.500162427 | 1.37E-27 | Positive |
| SCAF11 | GNG12-AS1 | 0.437947148 | 7.90E-21 | Positive |
| SCAF11 | ODF2-AS1 | 0.48371427 | 1.15E-25 | Positive |
| GZMB | MIR4435-2HG | 0.532040497 | 1.24E-31 | Positive |
| GZMA | LINC01614 | 0.45165808 | 3.34E-22 | Positive |
| SCAF11 | RNASEH2B-AS1 | 0.463689757 | 1.85E-23 | Positive |
| GZMA | LINC01711 | 0.645090904 | 4.39E-50 | Positive |
| CHMP4A | LINC01023 | 0.453859417 | 1.98E-22 | Positive |
| CHMP6 | LINC01023 | 0.424504181 | 1.53E-19 | Positive |
| GPX4 | LINC01023 | 0.46399 | 1.72E-23 | Positive |
| PJVK | DICER1-AS1 | 0.443714602 | 2.13E-21 | Positive |
| NLRC4 | LINC01915 | 0.529671176 | 2.57E-31 | Positive |
| PJVK | ZNF32-AS2 | 0.407075962 | 5.90E-18 | Positive |
| PLCG1 | ZNF32-AS2 | 0.411393719 | 2.43E-18 | Positive |
| ELANE | MIR100HG | 0.411120873 | 2.58E-18 | Positive |
| IL6 | MIR100HG | 0.455094096 | 1.48E-22 | Positive |
| SCAF11 | RMRP | 0.401230439 | 1.91E-17 | Positive |
| SCAF11 | GSN-AS1 | 0.506491456 | 2.33E-28 | Positive |
| SCAF11 | BACH1-IT2 | 0.466362236 | 9.56E-24 | Positive |
| SCAF11 | POU6F2-AS2 | 0.405057394 | 8.88E-18 | Positive |
| SCAF11 | LINC02569 | 0.4565887 | 1.03E-22 | Positive |
| IL18 | LINC02762 | 0.4149524 | 1.16E-18 | Positive |
| SCAF11 | ADAMTSL4-AS1 | 0.455785885 | 1.25E-22 | Positive |
| GSDMB | LINC01355 | 0.42864697 | 6.23E-20 | Positive |
| SCAF11 | SNHG16 | 0.432826095 | 2.48E-20 | Positive |
| SCAF11 | CRTC3-AS1 | 0.403256099 | 1.28E-17 | Positive |
| CHMP4A | GATA3-AS1 | 0.426608644 | 9.71E-20 | Positive |
| CHMP2A | LINC02560 | 0.423006862 | 2.11E-19 | Positive |
| SCAF11 | MED4-AS1 | 0.429416367 | 5.26E-20 | Positive |
| SCAF11 | RAP2C-AS1 | 0.428698229 | 6.16E-20 | Positive |
| SCAF11 | DENND6A-AS1 | 0.447027153 | 9.88E-22 | Positive |
| SCAF11 | ZDHHC20-IT1 | 0.486026352 | 6.24E-26 | Positive |
| SCAF11 | LINC01876 | 0.508006697 | 1.51E-28 | Positive |
| NLRC4 | LINC00882 | 0.474884691 | 1.12E-24 | Positive |
| GSDMB | ZNF32-AS1 | 0.439639156 | 5.39E-21 | Positive |
| PJVK | ZNF32-AS1 | 0.5127016 | 3.95E-29 | Positive |
| SCAF11 | TH2LCRR | 0.489028105 | 2.81E-26 | Positive |
| ELANE | LINC01638 | 0.413887986 | 1.45E-18 | Positive |
| GSDMB | LINC01004 | 0.4664605 | 9.33E-24 | Positive |
| PJVK | LINC01004 | 0.487739368 | 3.97E-26 | Positive |
| SCAF11 | DLEU2 | 0.409203852 | 3.82E-18 | Positive |
| CHMP4A | CHKB-DT | 0.409418613 | 3.65E-18 | Positive |
| SCAF11 | LINC02820 | 0.443412237 | 2.28E-21 | Positive |
| SCAF11 | LINC00630 | 0.635676576 | 3.00E-48 | Positive |
| GSDMB | HDAC2-AS2 | 0.428343332 | 6.66E-20 | Positive |
| PJVK | KLF3-AS1 | 0.423033273 | 2.10E-19 | Positive |
| CASP5 | LINC01943 | 0.471916892 | 2.38E-24 | Positive |
| GZMB | LINC01943 | 0.423818434 | 1.78E-19 | Positive |
| IRF1 | LINC01943 | 0.434434711 | 1.74E-20 | Positive |
| NLRC4 | LINC01943 | 0.456958791 | 9.47E-23 | Positive |
| NLRP3 | LINC01943 | 0.52217524 | 2.46E-30 | Positive |
| SCAF11 | PABPC4-AS1 | 0.551064378 | 2.95E-34 | Positive |
| SCAF11 | GSTCD-AS1 | 0.503145503 | 5.96E-28 | Positive |
| SCAF11 | OPA1-AS1 | 0.546331197 | 1.38E-33 | Positive |
| SCAF11 | FIRRE | 0.45170081 | 3.31E-22 | Positive |
| SCAF11 | LINC02042 | 0.46034378 | 4.18E-23 | Positive |
| PJVK | EXTL3-AS1 | 0.405420496 | 8.25E-18 | Positive |
| SCAF11 | LINC00641 | 0.491313194 | 1.53E-26 | Positive |
| CHMP4A | ZFHX2-AS1 | 0.588951178 | 5.16E-40 | Positive |
| GSDMB | ZFHX2-AS1 | 0.453812543 | 2.01E-22 | Positive |
| PJVK | ZFHX2-AS1 | 0.431827077 | 3.10E-20 | Positive |
| SCAF11 | LINC01376 | 0.445065895 | 1.56E-21 | Positive |
| SCAF11 | LINC01572 | 0.505438901 | 3.13E-28 | Positive |
| GPX4 | SNHG25 | 0.410511398 | 2.92E-18 | Positive |
| SCAF11 | MKNK1-AS1 | 0.471700203 | 2.52E-24 | Positive |
| SCAF11 | CARMN | 0.497051455 | 3.22E-27 | Positive |
| SCAF11 | CDC42-IT1 | 0.523070735 | 1.88E-30 | Positive |
| GSDMB | ZNF213-AS1 | 0.408656389 | 4.27E-18 | Positive |
| PJVK | ZNF213-AS1 | 0.482601581 | 1.54E-25 | Positive |
| PJVK | SEC24B-AS1 | 0.464285689 | 1.60E-23 | Positive |
| SCAF11 | SDCBP2-AS1 | 0.503285355 | 5.73E-28 | Positive |
| GSDMB | ARHGAP27P1-BPTFP1-KPNA2P3 | 0.406507156 | 6.62E-18 | Positive |
| PJVK | ARHGAP27P1-BPTFP1-KPNA2P3 | 0.4267945 | 9.33E-20 | Positive |
| NLRC4 | MED14OS | 0.459926909 | 4.63E-23 | Positive |
| NLRC4 | COL4A2-AS1 | 0.666676442 | 1.52E-54 | Positive |
| NLRP3 | COL4A2-AS1 | 0.414181197 | 1.37E-18 | Positive |
| CASP1 | LINC02195 | 0.425617276 | 1.20E-19 | Positive |
| IRF1 | LINC02195 | 0.578147548 | 2.70E-38 | Positive |
| SCAF11 | LINC00456 | 0.454397194 | 1.75E-22 | Positive |
| ELANE | MIR497HG | 0.605509544 | 8.91E-43 | Positive |
| NOD2 | MYOSLID | 0.44671053 | 1.06E-21 | Positive |
| IRF1 | ETV7-AS1 | 0.503597136 | 5.25E-28 | Positive |
| IL18 | LINC01836 | 0.44701494 | 9.91E-22 | Positive |
| SCAF11 | DHDDS-AS1 | 0.460786065 | 3.75E-23 | Positive |
| ELANE | LINC01119 | 0.406291805 | 6.92E-18 | Positive |
| IL6 | LINC01119 | 0.402508336 | 1.48E-17 | Positive |
| NLRP3 | LINC01119 | 0.444070896 | 1.96E-21 | Positive |
| NLRC4 | HMGA2-AS1 | 0.426034772 | 1.10E-19 | Positive |
| NLRC4 | SH3RF3-AS1 | 0.700927751 | 1.90E-62 | Positive |
| NLRP3 | SH3RF3-AS1 | 0.580648871 | 1.09E-38 | Positive |
| SCAF11 | LINC01126 | 0.459483894 | 5.15E-23 | Positive |
| NLRC4 | FENDRR | 0.513212166 | 3.41E-29 | Positive |
| SCAF11 | LINC-PINT | 0.417455637 | 6.88E-19 | Positive |
| GZMB | MEG9 | 0.452394996 | 2.81E-22 | Positive |
| GSDMB | NBR2 | 0.437565281 | 8.61E-21 | Positive |
| PJVK | SNHG20 | 0.424085501 | 1.68E-19 | Positive |
| SCAF11 | ITFG1-AS1 | 0.524391561 | 1.27E-30 | Positive |
| SCAF11 | RORA-AS1 | 0.533865972 | 7.07E-32 | Positive |
| NLRC4 | C1QTNF7-AS1 | 0.447325574 | 9.22E-22 | Positive |
| SCAF11 | MIRLET7A1HG | 0.534781593 | 5.33E-32 | Positive |
| NOD1 | MIR302CHG | 0.418998629 | 4.97E-19 | Positive |
| SCAF11 | MIR302CHG | 0.485354664 | 7.46E-26 | Positive |
| CHMP4A | KMT2E-AS1 | 0.40930396 | 3.74E-18 | Positive |
| GSDMA | LINC01527 | 0.487858144 | 3.84E-26 | Positive |
| NOD2 | LINC01527 | 0.438672464 | 6.71E-21 | Positive |
| NLRC4 | MAP3K20-AS1 | 0.529004704 | 3.15E-31 | Positive |
| NLRP3 | MAP3K20-AS1 | 0.429475228 | 5.20E-20 | Positive |
| GZMB | LINC02600 | 0.581911594 | 6.92E-39 | Positive |
| PJVK | OSGEPL1-AS1 | 0.483286 | 1.28E-25 | Positive |
| PJVK | C9orf163 | 0.418719263 | 5.27E-19 | Positive |
| SCAF11 | MCM3AP-AS1 | 0.512337151 | 4.39E-29 | Positive |
| SCAF11 | AP4B1-AS1 | 0.418985794 | 4.98E-19 | Positive |
| NLRC4 | GRK5-IT1 | 0.416521065 | 8.37E-19 | Positive |
| SCAF11 | GRK5-IT1 | 0.447393726 | 9.08E-22 | Positive |
| NLRP3 | VIM-AS1 | 0.429188742 | 5.53E-20 | Positive |
| CHMP4A | LINC00685 | 0.44204121 | 3.12E-21 | Positive |
| GSDMB | LINC00685 | 0.400047778 | 2.42E-17 | Positive |
| PJVK | LINC00685 | 0.43802066 | 7.77E-21 | Positive |
| CASP5 | PROSER2-AS1 | 0.405735337 | 7.74E-18 | Positive |
| SCAF11 | DCUN1D2-AS | 0.481231518 | 2.19E-25 | Positive |
| CASP5 | SOCAR | 0.404345178 | 1.03E-17 | Positive |
| NLRC4 | SOCAR | 0.474352128 | 1.29E-24 | Positive |
| NLRP3 | SOCAR | 0.476743505 | 6.99E-25 | Positive |
| SCAF11 | COX10-AS1 | 0.423074444 | 2.08E-19 | Positive |
| CASP1 | TTLL11-IT1 | 0.469820072 | 4.04E-24 | Positive |
| CASP5 | TTLL11-IT1 | 0.477476178 | 5.79E-25 | Positive |
| GSDMC | TTLL11-IT1 | 0.423399612 | 1.94E-19 | Positive |
| SCAF11 | NPTN-IT1 | 0.473526591 | 1.59E-24 | Positive |
| NLRC4 | TGFB2-AS1 | 0.651174738 | 2.64E-51 | Positive |
| NLRP3 | TGFB2-AS1 | 0.517151777 | 1.08E-29 | Positive |
| NLRP3 | MIR222HG | 0.415251468 | 1.09E-18 | Positive |
| SCAF11 | C1RL-AS1 | 0.467021615 | 8.12E-24 | Positive |
| CHMP6 | LINC02875 | 0.431618344 | 3.24E-20 | Positive |
| NLRP3 | GAPLINC | 0.466934441 | 8.29E-24 | Positive |
| NOD1 | PRC1-AS1 | 0.403657119 | 1.18E-17 | Positive |
| SCAF11 | PRC1-AS1 | 0.412148791 | 2.08E-18 | Positive |
| SCAF11 | TTC3-AS1 | 0.437833302 | 8.11E-21 | Positive |
| CHMP2A | MIR200CHG | 0.452865825 | 2.51E-22 | Positive |
| GZMA | LINC01705 | 0.426168988 | 1.07E-19 | Positive |
| SCAF11 | N4BP2L2-IT2 | 0.504026364 | 4.65E-28 | Positive |
| SCAF11 | LINC02156 | 0.494734114 | 6.05E-27 | Positive |

# Supplementary Figure legends.

## Figure S1. Prognostic value of the 9-pyroptosis-related-lncRNAs signature in patients of different clinical parameters.

(A-B) Kaplan-Meier curves for the overall survival of patients in the high- and low-risk groups in patients aged >65 and ≤65 of bladder cancer. (C-D) Kaplan-Meier curves for the overall survival of patients in the high- and low-risk groups in female and male patients of bladder cancer. (E-F) Kaplan-Meier curves for the overall survival of patients in the high- and low-risk groups in low and high grade of bladder cancer. (G-H) Kaplan-Meier curves for the overall survival of patients in the high- and low-risk groups in stage I-II and stage III-IV patients of bladder cancer.
